# Predicting the unpredictable: Prognostic and predictive power of FAPI-PET imaging in oncology: A systematic review and meta-analysis

**DOI:** 10.1007/s00259-025-07735-9

**Published:** 2026-01-27

**Authors:** Sajjad Sadeghpour, Atena Aghaee, Saba Sadeghpour, Alessio Rizzo, Daniele Antonio Pizzuto, Salvatore Annunziata, Giorgio Treglia, Ramin Sadeghi

**Affiliations:** 1 Nuclear Medicine Research Center, Mashhad University of Medical Sciences, Mashhad, Iran; 2 Madani Hospital, Gonabad University of Medical Sciences, Gonabad, Iran; 3 Department of Nuclear Medicine, Candiolo Cancer Institute, Torino, Italy; 4 Nuclear Medicine Unit, Fondazione Policlinico Universitario A. Gemelli IRCCS, Rome, Italy; 5 Division of Nuclear Medicine, Ente Ospedaliero Cantonale, Bellinzona, CH-6500 Switzerland; 6 Faculty of Biomedical Sciences, Università della Svizzera Italiana, Lugano, Switzerland; 7 Faculty of Biology and Medicine, University of Lausanne, Lausanne, Switzerland

**Keywords:** FAPI, PET, Cancer, Treatment response, Prognosis, Survival

## Abstract

**Objectives:**

In this systematic review, we evaluated the accuracy of baseline fibroblast activation protein inhibitor (FAPI)-based PET scans for predicting overall survival (OS), progression-free survival (PFS), and tumor response after treatment completion.

**Methods:**

We searched PubMed and Cochrane Library until August 2025 to identify studies that evaluated the prognostic and predictive value of FAPI-based PET scans parameters in the OS, PFS, and treatment response of cancer patients; all studies reporting these findings, whether as the main or a secondary finding, were included.

**Results:**

Of the forty-seven publications, including 1430 patients, various cancer types from breast to pancreatic cancer, were evaluated. The pooled hazard ratio (HR) indicated that patients with higher maximum standardized uptake value (SUVmax) were significantly associated with poor OS (HR: 2.36; 95% CI: 1.56–3.56, *p* < 0.011) and poor PFS (HR: 1.88; 95% CI: 1.36–2.60, *p* < 0.001). Patients with higher FAPI-avid tumor volume (MTV) were significantly associated with poorer OS (HR: 3.33; 95% CI: 1.99–5.56, *p* < 0.001) and PFS (HR: 2.42; 95% CI: 1.60–3.67, *p* < 0.001). High total lesion FAP expression (TLFAPI) was significantly associated with poorer OS (HR: 3.35; 95% CI: 2.05–5.50, *p* < 0.001) and PFS (HR: 2.45, 95% CI: 1.61–3.73, *p* < 0.001).

**Conclusion:**

Early evidence indicates that FAPI PET parameters, especially the baseline of MTV, SUVmax, and TLFAPI, can be proper prognostic markers of OS and PFS in cancers by imaging tumor stroma. In various cancers, FAPI uptake parameters change after treatment, or baseline levels are linked to treatment outcomes.

**Supplementary Information:**

The online version contains supplementary material available at https://doi.org/10.1007/s00259-025-07735-9.

## Introduction

Fibroblast activation protein (FAP) is a membrane-bound serine protease expressed by cancer-associated fibroblasts (CAFs) in the tumor stroma. CAFs are abundant in most epithelial tumors (often > 90% of cases) and facilitate matrix remodeling, tumor growth, invasion, and metastasis [1]. High stromal FAP expression is associated with aggressive tumor behavior. For example, colorectal cancers with FAP⁺ CAFs exhibit higher proliferation (Ki-67), mutated p53, and certain signaling pathways, which correlate with invasion and poor outcomes [2]. In many cancers, elevated FAP levels are linked to desmoplasia and resistance [3]. FAPI tracers bind FAP in vivo, resulting in high tumor uptake and minimal uptake in most normal tissues. This creates excellent tumor-to-background contrast, making FAPI-PET highly sensitive for detecting cancerous lesions [1].

Recent studies indicate that fibroblast activation protein inhibitor (FAPI)-based tracers, new PET radioligands targeting cancer-associated fibroblasts in the tumor microenvironment, can be utilized for disease detection, as many tumors rely on activated fibroblasts to maintain their viability and growth [4–6]. FAPI PET can be used for imaging different types of cancers and outperforms fluorine-18 fluorodeoxyglucose [^18^F]FDG PET/CT in some of these [7]. Its potential as a theranostic tool warrants special attention, whereas the prognostic and predictive value of baseline FAPI PET is still unclear.

The hypermetabolic state seen in tumoral cells after cancer treatment involves a marked increase in metabolic pathways, driven by adaptive responses to therapeutic stress. Treatments like chemotherapy, radiation, and targeted therapies cause oxidative stress, DNA damage, and nutrient shortages, leading cancer cells to boost glycolysis, glutaminolysis, and mitochondrial oxidative phosphorylation to satisfy increased energy and biosynthesis needs [8, 9], leading to increased cellular glucose uptake potentially noising [^18^F]FDG PET scans [10]. These effects can be seen in clinical practice, by an increased Standardized Uptake Value [11] in [^18^F]FDG PET scans [6].

To overcome these limitations of [^18^F]FDG PET, several studies used FAPI-based PET scans as an alternative molecular imaging method to assess treatment response in cancer patients and to predict the disease’s outcome [12–14].

On the other hand, using FAPI-based imaging can identify FAPI-avid lesions, suggesting the novel FAPI-based radioligand therapy agents, such as ^177^Lu, ^90^Y, and ^213^Bi, as a treatment option with promising and safe therapeutic results, in various cancers, such as thyroid cancer, breast cancer, and pancreatic ductal adenocarcinoma [15].

This systematic review aimed to evaluate the prognostic and predictive value of FAPI-based PET scans for overall survival (OS), progression-free survival (PFS), and treatment response assessment in various cancer patients receiving treatment.

## Methods

### Protocol

This systematic review was carried out according to a predefined protocol [16], using the PRISMA 2020 statement as a guideline, and in accordance with item 24 of the PRISMA checklist (Supplementary Fig. 1) [17]. This systematic review protocol was not pre-registered in registration domains.

The first step in this systematic review was to define the research question using the PICO framework (Population, Intervention, Comparator, Outcomes). The focus was on patients diagnosed with cancer (Population) who underwent FAPI-based PET scans (Intervention), with or without other imaging for comparison (Comparator). The primary outcomes were FAPI-based parameters as prognostic factors for OS or PFS. The secondary outcomes included parameters that could predict treatment response or differentiate between responders and non-responders.

### Literature search strategy, information sources, and eligibility criteria

After establishing the review question, a systematic search of PubMed/MEDLINE and Cochrane Library was conducted to identify studies evaluating the added value of FAPI-based imaging for cancer treatment response assessment. We used keywords such as “FAPI”, “FAP”, “PET” along with terms related to imaging methods like “cancer,” “tumor,” “neoplasm,” “malignancy,” as well as “response assessment,” “treatment response,” “therapeutic monitoring,” “therapy response”, “prognos*”, and “predict*”. Boolean operators (AND, OR) were employed to refine the search to relevant studies. No restrictions were placed on publication year. The reference lists of included studies were also examined for additional relevant articles. The most recent search was conducted in August 2025.

To maintain methodological rigor, reviews, letters, comments, editorials, preclinical studies, simulation studies, case series, and case reports were excluded from the systematic review. Only studies published in English met the eligibility criteria for inclusion.

### Study selection process and data collection

Titles and abstracts of all retrieved articles were reviewed to determine eligibility according to predefined inclusion and exclusion criteria. Relevant studies were systematically selected, and data were extracted using a standardized form to maintain consistency and accuracy. Information was gathered from the full texts.

For each included study, the extracted data encompassed: general study details (authors, publication year, country, study design, and funding sources); patient characteristics (sample size, age, sex distribution, clinical setting, cancer type, and stage of the disease); treatment details (type of therapy, line of therapy, timing of FAPI-based imaging in relation to the treatment); key characteristics of the index test (type of FAPI tracers); imaging assessment parameters (quantitative metrics); treatment response assessment (used criteria); and authors conclusion (main findings).

### Quality assessment (risk of bias assessment)

The quality and risk of bias (RoB) of the included studies were assessed using the Oxford University Centre for Evidence-Based Medicine (CEBM) critical appraisal tool for prognostic studies, evaluating the risk of bias of the studies in four domains of representativeness of cohort, follow-up, outcome measurement, and adjustment for confounding.

### Synthesis of results

Meta-analyses of hazard ratios for FAPI-based parameters regarding OS and PFS, along with the sensitivity and specificity of various parameters for distinguishing responders from non-responders, were conducted using a random-effects model (Der-Simonian and Laird method). This approach accounts for differences between studies, making it suitable for combining data from diverse sources. Heterogeneity was assessed with the Cochrane Q test, considering *p* < 0.05 as significant, and quantified using the I^2^ index, which shows the percentage of variability attributable to true heterogeneity rather than chance. The *I*^*2*^ statistics of 50–90% were considered substantial.

Publication bias was evaluated using funnel plots and Egger’s regression intercept. Funnel plots display the standard errors of the studies on the x-axis and their effect sizes on the y-axis. Any asymmetry suggests possible publication bias, which is quantified by Egger’s regression intercept (*p* < 0.05 indicates significant bias). The Duval–Tweedie trim-and-fill method corrects for this bias by iteratively removing smaller studies to restore symmetry, providing an adjusted pooled effect size to estimate the influence of publication bias. To address heterogeneity, subgroup analyses for each primary tumor type were conducted. Meta-analyses were performed with Comprehensive Meta-Analysis (version 2). The key prognostic indices examined included SUVmax, SUVmean, FAPI-avid Tumor Volume (MTV), Total Lesion FAP expression (TLFAPI), and Tumor-Background Ratio (TBR).

## Results

### Overview of included studies, patient and tumor characteristics

Forty-seven studies (published 2020–2025) met the inclusion criteria, comprising 1430 cancer patients (median ~ 27 per study, range 1–145). Most were prospective (*n* = 30), with the remainder retrospective (*n* = 17). The majority of studies were from China, with additional cohorts from Turkey, Germany, Israel, and one mixed international series. Tumor types were heterogeneous, including mixed solid tumors, breast cancer (notably invasive lobular), colorectal/rectal, pancreatic ductal adenocarcinoma, gastrointestinal stromal tumor (GIST), thyroid, urothelial, and other malignancies (Supplementary Table 1).

Most of the studies demonstrated low RoB in follow-up and outcome measurement domains. As many studies maintained a high overall RoB across domains, there remains a confounding limitation that warrants cautious interpretation of their findings (Supplementary Table 2).

### Imaging protocol and tracers

Most studies used ^68^Ga-labeled FAPI ligands in PET/CT or PET/MRI (predominantly FAPI-46 or FAPI-04), while others used ^18^F-labeled FAPI. [^18^F]FDG PET/CT was the principal comparator modality in most studies, serving as the reference metabolic scan.

### Baseline FAPI-derived parameters and response to treatment

Out of the forty-seven studies that met the inclusion criteria, 36 had enough numerical data on the selected parameters for the meta-analysis. Similar studies conducted at the same centers and during similar recruitment periods were evaluated for the meta-analysis, and those with more comprehensive data and larger sample sizes were chosen. This was done for similar studies [18–25], and [26–30].

Across the included studies, 56 quantitative baseline (pretreatment) PET parameters were extracted, including SUVmax, TBR, MTV, SUVpeak, TLFAPI, and SUVmean, as predictors of treatment response in cancer patients. Each parameter was pooled separately using a random-effects meta-analysis model. Baseline values of only TBR were significantly higher in non-responders compared to responders, with a mean difference (MD) of −1.29 (95% CI: −2.20 to −0.38, *p* = 0.005; Table 1, Supplemental Figs. 2–4). Heterogeneity was from 57.00% to 91.62% across various parameters.

**Table 1:** The mean difference of baseline FAPI-based PET scan quantitative parameters in responders vs. Non-responders, using Random-Effects analysis

Parameters	Mean Difference and 95% confidence interval					Heterogeneity			
	Number Studies	Point estimate	Lower limit	Upper limit	*P*-value	Q-value	df (Q)	*P*-value	I^2^
MTV	9	2.918	−6.713	12.548	0.553	46.659	8	< 0.001	82.854
SUVmax	15	−0.569	−2.185	1.047	0.490	167.246	14	< 0.001	91.629
SUVmean	10	−0.291	−1.211	0.628	0.535	34.663	9	< 0.001	74.035
SUVpeak	4	0.880	−0.642	2.402	0.257	6.978	3	0.073	57.007
TBR	8	−1.295	−2.206	−0.384	0.005	35.072	7	< 0.001	80.041
TLFAPI	10	23.745	−53.698	101.189	0.548	71.378	9	< 0.001	87.391

### Changes in FAPI-derived parameters during treatment and response

Across the included studies, 29 quantitative changes (pre- and post-treatment) PET parameters were extracted, encompassing SUVmax, TBR, MTV, SUVpeak, TLFAPI, and SUVmean as predictors of response to treatment in cancer patients. Each parameter was pooled separately using a random-effects meta-analysis model. Responders exhibited greater reductions in MTV (MD = 1.52, 95% CI [0.44, 2.60], *z* = 2.75, *p* = 0.005), SUVmax (MD = 1.78, 95% CI [1.41, 2.14], *z* = 9.55, *p* < 0.001), TBR (MD = 1.76, 95% CI [0.95, 2.57], *z* = 4.28, *p* < 0.001), SUVmean (MD = 2.00, 95% CI [1.02, 2.98], *z* = 4.00, *p* < 0.001) and TLFAPI (SMD = 2.30, 95% CI [0.78, 3.81], *z* = 2.97, *p* < 0.001). Heterogeneity ranged from 55.31% to 86.87% (Table 2, Supplemental Figs. 5–7).

**Table 2:** The mean difference of changes (before and after the therapy) of FAPI-based PET scan quantitative parameters in responders vs. Non-Responders, using Random-Effects analysis

Parameters	Number Studies	Mean Difference and 95% confidence interval				Heterogeneity			
		Point estimate	Lower limit	Upper limit	*P*-value	Q-value	df (Q)	*P*-value	I^2^
MTV	4	1.523	0.440	2.605	0.006	11.137	3	0.011	73.062
SUVmax	8	1.780	1.415	2.145	< 0.001	7.362	7	0.392	4.915
SUVmean	6	2.004	1.024	2.984	< 0.001	22.770	5	0.000	78.042
SUVpeak	2	3.488	0.850	6.126	0.010	7.072	1	0.008	85.859
TBR	4	1.766	0.957	2.574	< 0.001	6.714	3	0.082	55.316
TLFAPI	5	2.301	0.785	3.817	0.003	30.471	4	< 0.001	86.873

### Prognostic value for OS

**Fig. 1:**
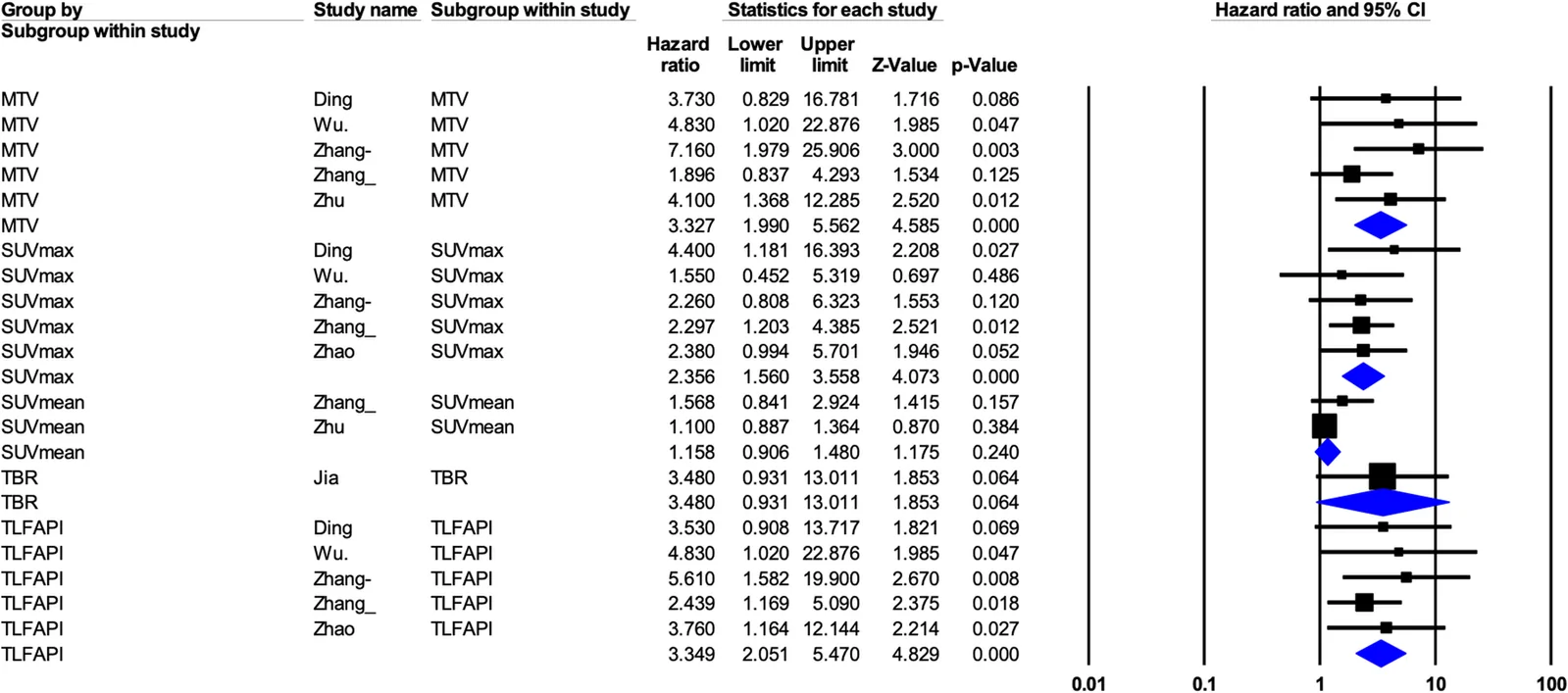
Prognostic Value of Baseline Parameters to Predict Overall Survival

Eighteen baseline FAPI-derived parameters were assessed across studies as the prognostic factor for OS. Random-effects models indicated that higher baseline MTV (HR = 3.33, 95% CI [1.99, 5.56], *z* = 4.59, *p* < 0.001), SUVmax (HR = 2.36, 95% CI [1.56, 3.56], *z* = 4.07, *p* < 0.001), and TLFAPI (HR = 3.35, 95% CI [2.05, 5.47], *z* = 4.83, *p* < 0.001) were associated with worse OS, whereas SUVmean (HR = 1.16, 95% CI [0.90, 1.48], *z* = 1.18, *p* = 0.24) and TBR (HR = 3.48, 95% CI [0.93, 13.01], *z* = 1.85, *p* = 0.064) showed nonsignificant trends (Fig. 1, Supplemental Fig. 8).

Potential publication bias among studies evaluating the prognostic value of FAPI-based PET-derived parameters for OS was assessed using visual inspection of the funnel plot and Egger’s regression test (Supplemental Fig. 9). The funnel plot appeared largely symmetrical, suggesting no evident small-study effects. This observation was supported by the results of Egger’s test, which demonstrated a non-significant intercept (t [3] = 2.22, *p* = 0.113, t [3] = 0.35, *p* = 0.748, t [7] = 1.11, *p* = 0.380, t [1] = 3.25, *p* = 0.203, and t [4] = 1.46, *p* = 0.219, for MTV, SUVmax, SUVmean, TBR, and TLFAPI respectively), indicating no statistically significant evidence of publication bias across the included studies Table 3.

**Table 3:** The pooled hazard ratio of the baseline FAPI-based PET scan quantitative parameters as prognostic factors for overall Survival, using Random-Effects analysis

Parameters	Number Studies	Effect size and 95% confidence interval				Heterogeneity			
		Point estimate	Lower limit	Upper limit	*P*-value	Q-value	df (Q)	*P*-value	I^2^
MTV	5	3.327	1.990	5.562	< 0.001	3.565	4	0.468	0.000
SUVmax	5	2.356	1.560	3.558	< 0.001	1.322	4	0.858	0.000
SUVmean	2	1.158	0.906	1.480	0.240	1.111	1	0.292	10.025
TBR	1	3.480	0.931	13.011	0.064	0.000	0	1.000	0.000
TLFAPI	5	3.349	2.051	5.470	< 0.001	1.608	4	0.807	0.000

### Prognostic value for PFS

**Fig. 2:**
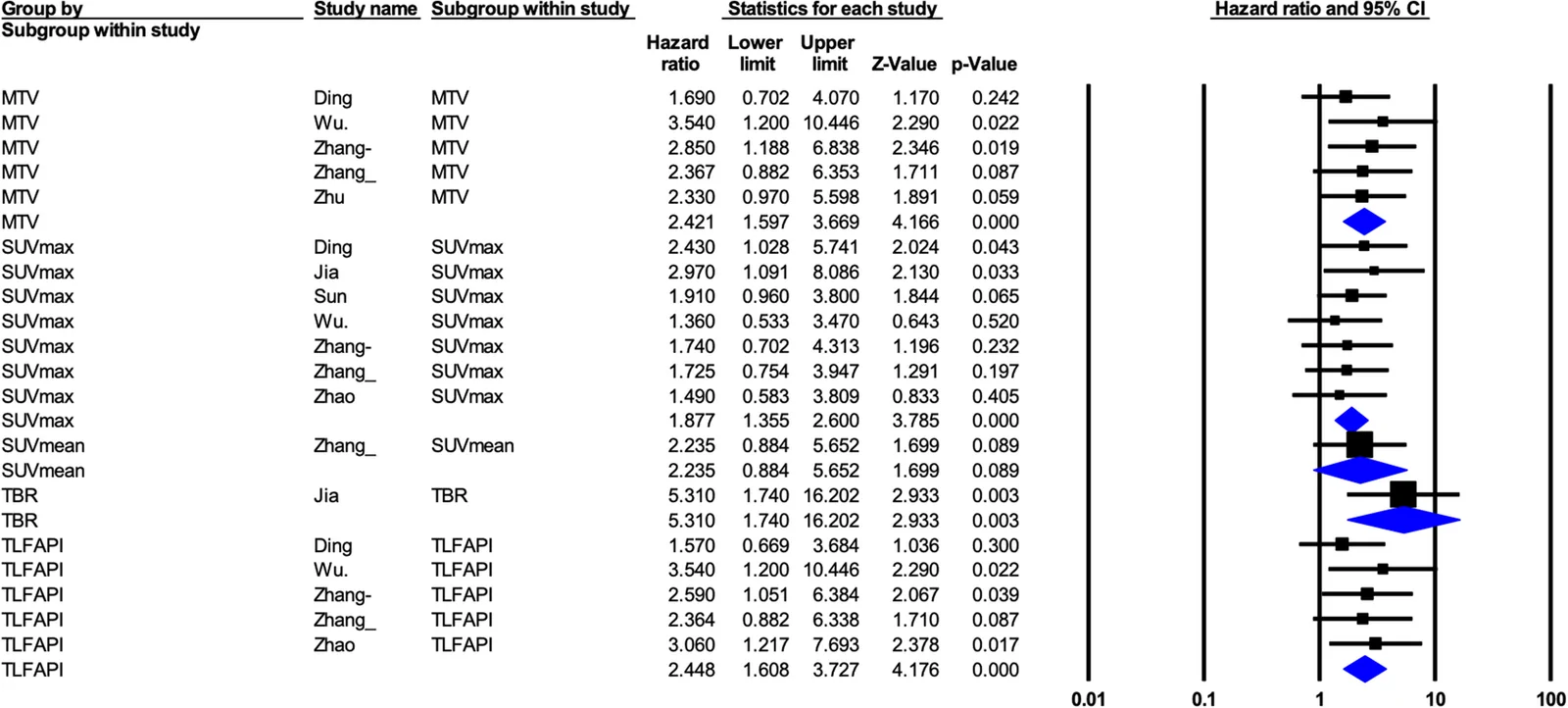
Prognostic Value of Baseline Parameters to Predict Progression-Free Survival

The overall nineteen studies evaluated FAPI parameters for PFS. Under random-effects models, higher baseline MTV (HR = 2.42, 95% CI [1.60, 3.67], *z* = 4.17, *p* < 0.001), SUVmax (HR = 1.88, 95% CI [1.35, 2.60], *z* = 3.79, *p* < 0.001), and TLFAPI (HR = 2.45, 95% CI [1.61, 3.73], *z* = 4.18, *p* < 0.001) significantly predicted worse PFS; SUVmean (HR = 2.24, 95% CI [0.88, 5.65], *z* = 1.70, *p* = 0.089) was marginally nonsignificant (Fig. 2, Supplemental Fig. 10).

Potential publication bias among studies evaluating the prognostic value of FAPI-based PET-derived parameters for PFS was assessed using visual inspection of the funnel plot and Egger’s regression test (Supplemental Fig. 11). The funnel plot appeared largely symmetrical, suggesting no evident small-study effects. This observation was supported by the results of Egger’s test, which demonstrated a non-significant intercept (t [2] = 1.02, *p* = 0.415, t [3] = 2.77, *p* = 0.07, t [7] = 0.828, *p* = 0.495, for MTV, SUVmax, and TLFAPI respectively), indicating no statistically significant evidence of publication bias across the included studies Table 4.

**Table 4:** The pooled hazard ratio of baseline FAPI-based PET scan quantitative parameters as prognostic factors for Progression-Free Survival, using Random-Effects analysis

Parameters	Number Studies	Effect size and 95% confidence interval				Heterogeneity			
		Point estimate	Lower limit	Upper limit	*P*-value	Q-value	df (Q)	*P*-value	I^2^
MTV	5	2.421	1.597	3.669	< 0.001	1.259	4	0.868	0
SUVmax	7	1.877	1.355	2.600	< 0.001	1.909	6	0.928	0
SUVmean	1	2.235	0.884	5.652	0.089	0.000	0	1.000	0
TBR	1	5.310	1.740	16.202	0.003	0.000	0	1.000	0
TLFAPI	5	2.448	1.608	3.727	< 0.001	1.733	4	0.785	0

### Diagnostic performance: sensitivity and specificity

Forty-three studies contributed to pooled sensitivity and specificity estimates for FAPI parameters in predicting treatment response. Random-effects models for sensitivity showed pooled performance of 0.76 (95% CI: [0.72, 0.80]), with considerable effects for most subgroups (e.g., MTV: 0.76, 95% CI: [0.62, 0.86]; SUVmax: 0.80, 95% CI: [0.67, 0.88]) except SUVpeak (0.62, 95% CI: [0.37, 0.82]), and SUVmean (0.73, 95% CI: [0.46, 0.90]). For specificity, the pooled random-effects were SUVmax (0.70, 95% CI: [0.54, 0.80]) and TLFAPI (0.75, 95% CI: [0.61, 0.85]) (Supplemental Figs. 12–15) Table 5 and 6.

**Table 5:** The pooled sensitivity of FAPI-based PET scan quantitative parameters as diagnostic factors for distinguishing responders vs. Non-Responders, using Random-Effect analysis

Parameters	Number Studies	Effect size and 95% confidence interval				Heterogeneity			
		Point estimate	Lower limit	Upper limit	*P*-value	Q-value	df (Q)	*P*-value	I^2^
ΔMTV	3	0.827	0.664	0.920	0.001	0.227	2	0.893	0.000
ΔSUVmax	5	0.794	0.638	0.894	0.001	0.926	4	0.921	0.000
ΔSUVmean	2	0.872	0.703	0.951	< 0.001	0.299	1	0.585	0.000
ΔSUVpeak	3	0.776	0.582	0.896	0.007	0.159	2	0.924	0.000
ΔTBR	3	0.759	0.553	0.889	0.016	1.500	2	0.472	0.000
ΔTLFAPI	2	0.799	0.527	0.934	0.033	0.069	1	0.793	0.000
MTV	5	0.761	0.618	0.863	0.001	0.762	4	0.943	0.000
SULpeak	1	0.663	0.555	0.756	0.004	0.000	0	1.000	0.000
SUVmax	6	0.798	0.671	0.884	< 0.001	4.134	5	0.530	0.000
SUVmean	2	0.736	0.466	0.899	0.084	0.724	1	0.395	0.000
SUVpeak	2	0.621	0.370	0.821	0.345	0.418	1	0.518	0.000
TBR	5	0.831	0.694	0.915	< 0.001	3.669	4	0.453	0.000
TLFAPI	4	0.768	0.562	0.895	0.013	3.729	3	0.292	19.547

**Table 6:** The pooled specificity of FAPI-based PET scan quantitative parameters as diagnostic factors for distinguishing responders vs. Non-Responders, using Random-Effects analysis

Parameters	Number Studies	Effect size and 95% confidence interval				Heterogeneity			
		Point estimate	Lower limit	Upper limit	*P*-value	Q-value	df (Q)	*P*-value	I^2^
ΔMTV	3	0.895	0.790	0.951	< 0.001	1.233	2	0.540	0.000
ΔSUVmax	5	0.898	0.795	0.952	< 0.001	1.563	4	0.815	0.000
ΔSUVmean	2	0.745	0.615	0.843	0.001	0.024	1	0.877	0.000
ΔSUVpeak	3	0.888	0.747	0.955	< 0.001	1.166	2	0.558	0.000
ΔTBR	3	0.849	0.692	0.933	< 0.001	2.118	2	0.347	5.554
ΔTLFAPI	2	0.867	0.629	0.962	0.006	0.986	1	0.321	0.000
MTV	5	0.605	0.461	0.732	0.151	4.457	4	0.348	10.250
SULpeak	1	0.593	0.403	0.758	0.339	0.000	0	1.000	0.000
SUVmax	6	0.700	0.544	0.821	0.014	6.404	5	0.269	21.924
SUVmean	2	0.799	0.571	0.923	0.014	0.050	1	0.822	0.000
SUVpeak	2	0.749	0.520	0.892	0.034	0.067	1	0.796	0.000
TBR	5	0.690	0.549	0.803	0.010	1.902	4	0.754	0.000
TLFAPI	4	0.755	0.614	0.856	0.001	1.832	3	0.608	0.000

## Discussion

### Responder vs. non-responders in baseline parameters

When evaluated with the random-effects model, the predictive value of baseline FAPI-based PET parameters provided subtle insights. Among all assessed parameters, only the TBR remained statistically significant (effect size = − 1.295, 95% CI: − 2.206 to − 0.384, *p* = 0.005), indicating that higher TBR values at baseline are consistently associated with poorer treatment responses across different study populations and tumor types. This underscores TBR’s potential as a reliable prognostic biomarker, less influenced by variations in study design, imaging techniques, or patient characteristics. Conversely, parameters like MTV, SUVmax, SUVmean, SUVpeak, and TLFAPI did not reach significance in the random-effects model, although some were significant under fixed-effect assumptions.

In tumor biology, an elevated TBR indicates increased FAP expression in CAFs, which are instrumental in stromal remodeling and desmoplasia, especially in pancreatic ductal adenocarcinoma (PDAC) and esophageal squamous cell carcinoma (ESCC) [31, 32]. In PDAC, CAFs contribute to chemoresistance by secreting extracellular matrix (ECM) components like collagen, which impairs drug penetration and promotes hypoxia, particularly during chemotherapy [33]. Similarly, in ESCC, high FAP levels (high FAPI uptake post-treatment) are associated with immunosuppressive microenvironments, leading to reduced efficacy of immunotherapy [31]. On the other hand, parameters such as MTV, SUVmax, SUVmean, SUVpeak, and TLFAPI did not show significance in the random-effects model, despite some being significant under fixed-effect assumptions. This indicates that the predictive value of these measures may vary depending on specific contexts and be affected by heterogeneity in patient groups, cancer types and subtypes, treatment protocols, imaging procedures, or measurement techniques. For example, SUV metrics can be influenced by imaging protocols and only reflect localized hot spots. Meanwhile, MTV/TLFAPI measures overall tumor-stroma burden but depends on lesion size and segmentation. In breast cancer treated with neoadjuvant chemotherapy (NAC), a high baseline SUVmax indicates poor response to therapy and possibly aggressive CAF-mediated invasion. However, variability exists due to differences in hormone receptor status that affect stromal activation [34]. Evidence shows that FAPI SUV or MTV correlates with CAF markers (e.g., α-SMA, vimentin), but their predictive power can vary by cancer type. In pancreatic cancer, for example, higher baseline MTV (which reflects larger FAP⁺ volume) is associated with poorer survival [35]. Overall, the significance of TBR suggests that the normalized contrast of tumor uptake is a consistent prognostic feature that is less affected by protocol differences.

The heterogeneity index across metrics was substantial to considerable. This was more pronounced for SUVmax. These high heterogeneities can be attributed to various factors, including tumor heterogeneity across different cancers, different treatment regimens, response definitions, cancer types and subtypes, imaging procedures, measurement techniques, desmoplastic reactions, radiopharmaceuticals, imaging acquisition and protocol, reference standards, and other pathophysiological conditions. On the other hand, parameters such as SUVmax can be highly noise-dependent, further contributing to heterogeneity. Or MTV can be ROI-dependent, which varies across readers.

### Responders vs. non-responders, before and after the treatment

The pooled analysis of parameter changes before and after treatment revealed that all evaluated FAPI-based parameters significantly decreased in responders compared to non-responders. SUVmax (effect size = 1.780, 95% CI: 1.415–2.145, *p* < 0.001) was the most stable and consistently significant metric, highlighting its reliability in distinguishing responders from non-responders despite variability across studies. Likewise, SUVmean (effect size = 2.004, 95% CI: 1.024–2.984, *p* < 0.001) and TLFAPI (effect size = 2.301, 95% CI: 0.785–3.817, *p* = 0.003) both showed strong predictive ability, indicating that both average uptake and total lesion burden decrease notably with successful treatment. SUVpeak (effect size = 3.488, 95% CI: 0.850–6.126, *p* = 0.010) and TBR (effect size = 1.766, 95% CI: 0.957–2.574, *p* < 0.001) also supported these results, although their broader confidence intervals suggest greater variability across studies. Notably, MTV (effect size = 1.523, 95% CI: 0.440–2.605, *p* = 0.006) was also significant, indicating volumetric changes are relevant but may be more heterogeneous.

Biologically, these reductions can be related to therapy-induced CAF apoptosis and ECM breakdown. In gastric adenocarcinoma treated with NAC, decreasing SUVmax and MTV indicate less FAP-driven fibrosis, improving drug delivery and immune cell infiltration [36]. In responders, all major FAPI parameters (SUVmax, SUVmean, SUVpeak, MTV, TLFAPI, TBR) significantly decreased after treatment, while non-responders showed little change. This is expected: effective therapy (chemotherapy, radiation, or immunotherapy) kills tumor cells and associated CAFs, lowering FAP expression and tracer uptake. For example, Zhu et al. found that in pancreatic cancer patients, larger reductions in SUVmax, MTV, and TLFAPI during chemotherapy were associated with a better response. Among these metrics, SUVmax and SUVmean proved to be particularly reliable: post-treatment decreases in peak and average uptake indicated reduced metabolic activity in the most aggressive tumor sites. Similarly, MTV and TLFAPI (the volumetric extent of FAP⁺ tissue) decreased as the tumor shrank. In colorectal cancer with TGF-β inhibition and immunotherapy, falling TLFAPI values indicate disrupted communication between CAFs and tumor cells, which reduces metastasis by lowering cytokine signaling, such as CXCL12 [37, 38].

Regarding heterogeneity, heterogeneous ROI can affect SUVmean and MTV, and thus TLFAPI, which demonstrated considerable heterogeneity. On the other hand, SUVmax, despite its noise dependence, can be more reliable, more reproducible, and less heterogeneous before and after treatment, in each individual, as shown by its lowest *I*^*2*^ index.

In a true responder, treatment often leads to cell death or stromal changes that reduce FAPI binding. In many cases, that results in a relatively large drop in the hottest voxel, and that drop may be similar across different tumor types, treatments, and centers. This consistent direction of change across heterogeneous studies leads to smaller between-study variance in ΔSUVmax, especially when the magnitude of the drop is large relative to technical noise.

ΔSUVmean, ΔFTV, ΔTLFAPI, and ΔTBR require segmenting the entire lesion, re-drawing VOIs at baseline and after treatment, and carefully matching them. Differences in segmentation methods, background ROI definitions, or lesion delineation across studies will lead to substantial variability. In contrast, ΔSUVmax is a single-voxel statistic; it does not require volumetric segmentation or the precise delineation of lesion boundaries. That dramatically reduces the methodological sources of heterogeneity.

For volumetric metrics, noise, partial-volume effects, motion differences, and misregistration between baseline and follow-up scans may strongly influence the measured change, especially if the tumor shrinks or changes shape. This variability adds to heterogeneity. For SUVmax, some of this noise may cancel out. If acquisition parameters are similar and the hottest voxel remains within the same tumor region, the relative change tends to be more robust than volume-based measures across different centers.

### Baseline parameters to predict OS

The pooled HRs showed that several FAPI-based PET baseline parameters have strong and consistent prognostic value for OS. Notably, MTV and TLFAPI were the most prominent predictors, with HRs of 3.327 (95% CI: 1.990–5.562, *p* < 0.001) and 3.349 (95% CI: 2.051–5.470, *p* < 0.001), respectively. These results suggest that patients with higher baseline MTV or TLFAPI are more likely to have significantly worse survival outcomes, highlighting the importance of volumetric and total metabolic burden measures. SUVmax also showed a meaningful association with OS (HR = 2.356, 95% CI: 1.560–3.558, *p* < 0.001), suggesting that higher peak tracer uptake may reflect more aggressive disease and a poorer prognosis. Conversely, SUVmean (HR = 1.158, 95% CI: 0.906–1.480, *p* = 0.240) and TBR (HR = 3.480, 95% CI: 0.931–13.011, *p* = 0.064) did not reach statistical significance, possibly due to limited studies and variability in imaging protocols or tumor features. Importantly, heterogeneity was minimal across most parameters (I² = 0% for MTV, SUVmax, and TLFAPI), confirming the consistency and robustness of these findings across different cohorts.

Biologically, high MTV and TLFAPI in PDAC are linked to extensive stromal desmoplasia, which promotes angiogenesis and metastasis through FAP-mediated pathways, resulting in poor OS after chemotherapy [19]. These findings are biologically plausible: a larger amount of FAP-expressing stroma indicates a more extensive desmoplastic and tumor environment, suggesting faster disease progression. MTV, reflecting the volumetric extent of FAPI-avid tumor burden, indicates that patients with higher MTV at baseline face over three times the risk of death compared to those with lower values. Similarly, TLFAPI, which integrates both tumor volume and uptake intensity, underscores its robustness in capturing overall disease aggressiveness. SUVmax also has prognostic value, likely because very high FAPI uptake highlights highly active cancer regions. In contrast, baseline SUVmean and TBR showed less consistent prognostic significance, possibly due to limited data or variability in background sampling. SUVmax, a measure of peak tracer uptake intensity, also performed strongly, suggesting it effectively identifies focal areas of high fibroblast activity linked to adverse outcomes. These parameters outperformed SUVmean and TBR, likely because MTV and TLFAPI account for the heterogeneous, multifocal nature of tumors in mixed cancer cohorts. In contrast, SUVmax provides a straightforward indicator of metabolic hotspots. SUVmean, averaging uptake across lesions, may dilute prognostic signals by including less aggressive regions, which may explain its lack of significance.

The low heterogeneity indicates high consistency in effect sizes across the included studies, despite the mixed cancer types. Low heterogeneity suggests that the prognostic utility of these parameters is not substantially influenced by inter-study variations in patient populations, imaging protocols, or cancer-specific biology, enhancing the generalizability of the results. For SUVmean, moderate heterogeneity may stem from the smaller number of studies, potentially reflecting differences in measurement thresholds or tumor types. The absence of heterogeneity for TBR is uninformative due to the single-study design.

### Baseline parameters to predict PFS

The pooled analysis highlighted that several baseline FAPI-based PET parameters promise prognostic value for PFS. Notably, MTV and TLFAPI showed the strongest and most consistent associations, with HRs of 2.421 (95% CI: 1.597–3.669, *p* < 0.001) and 2.448 (95% CI: 1.608–3.727, *p* < 0.001), respectively. These results indicate that patients with higher baseline volumetric or total metabolic tumor burden have more than double the risk of disease progression compared to those with lower values. SUVmax also demonstrated a significant predictive association with PFS (HR = 1.877, 95% CI: 1.355–2.600, *p* < 0.001), suggesting that tumors with higher peak tracer uptake are more likely to exhibit earlier progression. Interestingly, TBR, though evaluated in a single study, showed a markedly elevated HR (5.310, 95% CI: 1.740–16.202, *p* = 0.003), implying that contrast between tumor and background uptake may also serve as a sensitive marker of aggressive disease biology. In contrast, SUVmean did not reach statistical significance (HR = 2.235, 95% CI: 0.884–5.652, *p* = 0.089), likely reflecting limited data availability. Importantly, heterogeneity was negligible across all parameters (I² = 0%), indicating strong consistency and reproducibility of these findings among included studies.

In the view of pathophysiology, in ESCC undergoing chemoradiotherapy, high MTV indicates FAP-upregulated stromal barriers that promote progression by limiting radiation-induced apoptosis [39]. In neuroendocrine neoplasms, discordant FDG+/FAPI- lesions suggest aggressive CAF phenotypes, which are associated with shorter PFS due to increased ECM stiffness and invasion [40].

### Baseline parameters sensitivity to diagnose responders vs. non-responders

FAPI-based PET parameters show high and consistent sensitivity in distinguishing treatment responders from non-responders. Across all parameters, the pooled sensitivity remains strong, with an overall value of 76% (95% CI: 71.8–79.8%, *p* < 0.001), indicating that approximately 76% of responders are correctly identified using these imaging biomarkers. Among individual indices, both baseline and post-treatment changes (Δ-parameters) perform comparably well. The highest sensitivities are observed for ΔSUVmean (87.2%, 95% CI: 70.3–95.1%, *p* < 0.001) and ΔMTV (82.7%, 95% CI: 66.4–92.0%, *p* = 0.001), suggesting that reductions in mean uptake and metabolic tumor volume after therapy most effectively differentiate responders. Similarly, ΔSUVmax (79.4%, *p* = 0.001), ΔTLFAPI (79.9%, *p* = 0.033), and ΔTBR (75.9%, *p* = 0.016) also demonstrate good diagnostic sensitivity, supporting their value as early response markers. Baseline parameters such as SUVmax (79.8%, *p* < 0.001), TBR (83.1%, *p* < 0.001), and MTV (76.1%, *p* = 0.001) also show comparable discriminative performance. Importantly, all analyses display negligible heterogeneity (I² = 0%), reflecting excellent consistency across studies and imaging protocols.

Biologically, in breast cancer with NAC, high sensitivity of ΔSUVmean results from CAF deactivation, which reduces FAP expression and correlates with a pathologic complete response by decreasing stromal support for tumor growth [34].

### Baseline parameters specificity to diagnose responders vs. non-responders

Considering the specificity of FAPI-based PET parameters for distinguishing treatment responders from non-responders, the overall pooled specificity was 75.4% (95% CI: 71.4–79.0%, *p* < 0.001), indicating that about 75% of non-responders were correctly identified, confirming the strong discriminative ability of these imaging biomarkers. Among the change-based (Δ) parameters, ΔSUVmax (89.8%, *p* < 0.001), ΔMTV (89.5%, *p* < 0.001), and ΔSUVpeak (88.8%, *p* < 0.001) showed the highest specificities, highlighting their reliability in accurately excluding non-responders after treatment. ΔTLFAPI (86.7%, *p* = 0.006) and ΔTBR (84.9%, *p* < 0.001) also performed very well, indicating that changes in total lesion activity and tumor-to-background contrast are dependable markers of treatment inefficacy. Baseline parameters such as SUVmax (70.0%, *p* = 0.014), TLFAPI (75.5%, *p* = 0.001), and TBR (69.0%, *p* = 0.010) maintained moderate yet significant specificity, emphasizing their importance in pretreatment prognosis. Notably, heterogeneity across studies was low (I² = 20.4%), reflecting high consistency and reproducibility across imaging centers and cancer types.

Pathophysiologically, in rectal cancer treated with neoadjuvant radiotherapy and immunochemotherapy, high ΔSUVmax specificity indicates persistent CAF activity in non-responders, which maintains ECM integrity and promotes resistance through TGF-β signaling [41].

### Methodological Considerations, limitations of the review, and future directions

Tumor and microenvironment heterogeneity are confounders. Different histologies have different CAF densities: pancreatic and breast cancers tend to have dense desmoplasia, colorectal cancers often have moderate desmoplasia, whereas hematologic malignancies may have variable stroma. FAPI uptake will reflect these differences. In addition, therapy itself can alter FAP expression (e.g., radiation/fibrosis can increase benign FAPI uptake).

The present study has several limitations that should be disclosed. The small sample sizes and the retrospective design of most included studies limit the strength of the conclusions. Several studies lacked complete data on histopathology correlation, survival outcomes, or different definitions of response. Additionally, only a few studies provided quantitative performance metrics and direct comparisons with standard imaging modalities. Another limitation was that many studies did not include head-to-head comparisons with FDG. We acknowledge that the study included heterogeneous cancer subtypes, various FAPI-based tracers, and examined FAPI PET across a range of indications and time points. All of these affect the generalizability of the findings and potentially impact the robustness of prognostic performance estimates for PET imaging.

Larger, prospective studies using standardized imaging parameters and unified response criteria with harmonized protocols (same tracer, imaging times, and response criteria) are needed. Further exploration of FAPI’s prognostic value and integration with theranostic approaches (e.g., ²¹³Bi-FAPI) could broaden its clinical application. Studies should also report on reproducibility and observer agreement to support clinical adoption. Further research on the potential capability of FAPI-based imaging in response assessment of treatment, such as immunotherapy, particularly immune checkpoint inhibitors and TGF-βR pathway inhibition, can be of interest.

## Conclusion

Early evidence indicates that FAPI PET parameters, especially the baseline of MTV, SUVmax, and TLFAPI, can be sensitive prognostic markers of OS and PFS in certain cancers by imaging tumor stroma. In different tumors, FAPI uptake parameters at baseline evaluation or their changes after treatment have been linked to survival and treatment outcomes. This ability makes them into a potentially valuable tool for prognosis and treatment outcome prediction. However, the current evidence is limited and is based on preliminary results, and future larger studies are needed to confirm these preliminary findings.

## Supplementary Information

Below is the link to the electronic supplementary material.

Supplementary Material 1 (Characteristics of the included articles. DOCX 50.8 KB)

Supplementary Material 2 (Risk-of-bias of the included studies using the Oxford University for CEBM checklist for Prognostic Studies. DOCX 41.8 KB)

Supplementary file 3 (PRISMA flow diagram. PNG 183 KB)

Supplementary Material 3 (TIF 2.92 MB)

Supplementary Material 4 (The Forest Plot of Baseline FAPI-based PET scan Quantitative Parameters in Responders vs. Non-responders, Using Random-Effects Analysis. PNG 1.16 MB)

Supplementary Material 5 (The Forest Plot of Baseline FAPI-based PET scan Quantitative Parameters in Responders vs. Non-responders, Using Random-Effects Analysis, in Subgroups of Various Tumor Types, **A**: MTV, **B**: SUVmax, **C**: SUVmean, **D**: SUVpeak, **E**: TBR, **F**: TLFAPI. PNG 1.31 MB)

Supplementary Material 6 (Funnel plot assessing publication bias for studies evaluating the Baseline FAPI-based PET-derived Parameters in Responders vs. Non-responders. **A**: MTV, **B**: SUVmax, **C**: SUVmean, **D**: SUVpeak, **E**: TBR, **F**: TLFAPI. PNG 499 KB)

Supplementary Material 7 (The Forest Plot of Changes of FAPI-based PET scan Quantitative Parameters in Responders vs. Non-responders, Using Random-Effects Analysis. PNG 592 KB)

Supplementary Material 8 (The Forest Plot of Changes of FAPI-based PET scan Quantitative Parameters in Responders vs. Non-responders, Using Random-Effects Analysis, in Subgroups of Various Tumor Types, **A**: MTV, **B**: SUVmax, **C**: SUVmean, **D**: SUVpeak, **E**: TBR, **F**: TLFAPI.PNG 938 KB)

Supplementary Material 9 (Funnel plot assessing publication bias for studies evaluating the Changes of FAPI-based PET-derived Parameters in Responders vs. Non-responders. **A**: MTV, **B**: SUVmax, **C**: SUVmean, **D**: TBR, **E**: TLFAPI. 508 KB)

Supplementary Material 10 (Prognostic Value of Baseline Parameters to Predict Overall Survival, in Subgroups of Various Tumor Types, **A**: MTV, **B**: SUVmax, **C**: SUVmean, **D**: TBR, **E**: TLFAPI. PNG 737 KB)

Supplementary Material 11 (Funnel plot assessing publication bias for studies Predicting Overall Survival by FAPI-based PET-derived Parameters. **A**: MTV, **B**: SUVmax, **C**: TLFAPI (as the SUVmean and TBR had fewer than 3 articles, the publication bias was not assessed for them). PNG 295 KB)

Supplementary Material 12 (Prognostic Value of Baseline Parameters to Predict Progression-Free Survival, in Subgroups of Various Tumor Types, **A**: MTV, **B**: SUVmax, **C**: TLFAPI. PNG 500 KB)

Supplementary Material 13 (Funnel plot assessing publication bias for studies Predicting Progression-Free Survival by FAPI-based PET-derived Parameters. **A**: MTV, **B**: SUVmax, **C**: TLFAPI (as the SUVmean and TBR had fewer than 3 articles, the publication bias was not assessed for them). PNG 463 KB)

Supplementary Material 14 (The Forest Plot of Baseline FAPI-based PET scan Quantitative Parameters in Sensitivity to Diagnose Responders vs. Non-responders, Using Random-Effects Analysis, in Subgroups of Various Tumor Types, **A**: ∆MTV, **B**: ∆SUVmax, **C**: ∆SUVmean, **D**: ∆SUVpeak, **E**: ∆TBR, **F**: MTV, **G**: SUVmax, **HH**: SUVmean, **I**: SUVpeak, **J**: TBR, **K**: TLFAPI. PNG 1.14 MB)

Supplementary Material 15 (Funnel plot assessing publication bias for studies evaluating the FAPI-based PET-derived Parameters Sensitivity to Diagnose Responders vs. Non-responders. **A**: ∆MTV, **B**: ∆SUVmax, **C**: ∆SUVpeak, **D**: ∆TBR, **E**: MTV, **F**: SUVmax, **G**: TBR, **H**: TLFAPI (as the ∆SUVmean, ∆TLFAPI, SULpeak, SUVmean, and SUVpeak had fewer than three articles, the publication bias was not assessed for them). PNG 689 KB)

Supplementary Material 16 (The Forest Plot of Baseline FAPI-based PET scan Quantitative Parameters in Specificity to Diagnose Responders vs. Non-responders, Using Random-Effects Analysis, in Subgroups of Various Tumor Types, **A**: ∆MTV, **B**: ∆SUVmean, **C**: ∆SUVmax, **D**: ∆SUVpeak, **E**: ∆TBR, **F**: MTV, **G**: SUVmax, **H**: SUVmean, **I**: SUVpeak, **J**: TBR, **K**: TLFAPI. PNG 0.97 MB)

Supplementary Material 17 (Funnel plot assessing publication bias for studies evaluating the FAPI-based PET-derived Parameters Specificity to Diagnose Responders vs. Non-responders. **A**: ∆MTV, **B**: ∆SUVmax, **C**: ∆SUVpeak, **D**: ∆TBR, **E**: MTV, **F**: SUVmax, **G**: TBR, **H**: TLFAPI (as the ∆SUVmean, ∆TLFAPI, SULpeak, SUVmean, and SUVpeak had fewer than three articles, the publication bias was not assessed for them). PNG 714 KB)

## Data Availability

All data generated or analysed during this study are included in the manuscript. The datasets extracted from the included studies and the analysis code are available from the corresponding author upon reasonable request. All original source data are available from the cited publications.
