# Supplementary material for: Predicting the unpredictable: Prognostic and predictive power of FAPI-PET imaging in oncology: A systematic review and meta-analysis
Source: Eur J Nucl Med Mol Imaging. 2026 Jan 27;53(5):2927–38. doi: 10.1007/s00259-025-07735-9 (PMC13649892; doi:10.1007/s00259-025-07735-9)
Supplement: Supplementary file 1 [file 259_2025_7735_MOESM1_ESM.docx]

***Supplementary Table 1. Characteristics of the included articles.***

| Study | Country | study design | cancer type | sample size | age | Sex distribution | Type of therapy | FAPI radiotracer | Quantitative metrics | Criteria used | Summary of main findings |
| --- | --- | --- | --- | --- | --- | --- | --- | --- | --- | --- | --- |
| Backhaus et al. (1) | Germany | retrospective | breast | 13 | 47 | All F | chemotherapy | 68Ga-FAPI-46 | SUVmax, TBRmax/max | pathologic response | FAPI PET/MRI outperformed MRI in classifying response. |
| Cermik et al. (2) | Turkey | prospective | Rectum | 1 | 48 | 1 M | chemotherapy. | 68Ga-FAPI-04. | SUVmax, TBR. | Visual assessment (PR, SD, PD). | NS |
| Helisch et al. (3) | Germany | retrospective | Colon, breast, anal, prostate | 6 | 16-77 | 4 F, 2 M | 213Bi-FAPI RPT (+ pembrolizumab in 4/6). | 68Ga-FAPI-46 (diagnostic), 213Bi-FAPI-46 (therapy). | TLFAPI | Visual assessment (PR, SD, PD). | NS |
| Dong et al. (4) | China | phase 2 trial | Locally advanced esophageal SCC | 32 | 65 | 29 M, 3 F | Neoadjuvant chemotherapy | 18 F-FAPI (AIF-NOTA-FAPI-04) | SUVmax, SUVpeak, SUVmean, TBR, MTV, TLF | Pathologic response | FAPI PET parameters post-treatment predict pathologic response to treatment. |
| Chen et al. (5) | China | prospective | Pancreatic cancer, gastric cancer | 13 | 51 | 7 M, 6F | NS | 68Ga-FAPI-04 | K 1, k2, k3, k4, total distribution volume, SNR, CNR | NS | NS |
| Wu et al. (6) | China | retrospective | Recurrent or metastatic GISTs | 27 | 60 | 18 M, 9 F | Targeted therapy (imatinib, sunitinib, regorafenib, ripretinib). | 18F-FAPI-42 | SUVmean, SUVmax, SULpeak, TBR, MTV, TLFAPI. | RECIST 1.1 | FAPI PET/CT complements FDG PET/CT for response prediction in GISTs. |
| Novruzov et al. (7) | Germany, Chile | prospective | PDAC | 4 | 70 | 6M, 1 F | Chemoradiotherapy | 18F-FAPI-74 | SUVmax, SUVmean, TBR. | NS | FAPI shows promise for PDAC restaging. |
| Xiong et al. (8) | China | prospective | liver, stomach, pancreas, intestine | 22 | 57 | 30 M, 12 F | NS | 18F-FAPI-42 | SUVmax, TBR. | NS | Modified treatment in 40.48% of patients vs. 4.8% with FDG. |
| Li et al. (9) | China | prospective | PDAC | 48 | NS | NS | Systemic Treatment | 18F-FAPI-04 | SUVmax, TBR. | NS | NS |
| Chen et al. (10) | China | retrospective | pancreas | 9 | 53 | 6 M, 3 F | Chemotherapy | 68Ga-FAPI-04 | SUVmax, SUVmean, TBRmean, TBRmax, total distribution volume | NS | TBRmean reflects FAP receptor density better than SUV. A promising tool for treatment response assessment. |
| Li et al. (11) | China | prospective | pancreas | 28 | 61 | NS | Chemotherapy ± anti-PD-1 immunotherapy | 18F-FAPI-04 | SUVmax, SUVmean, T/B ratios, MTV, TLF | CAP pathologic response score | Greater decreases in FAPI uptake post-treatment predicted better pathologic response and survival. |
| Zhu et al. (12) | China | prospective | PDAC | 17 | 60 | 9 M, 8 F | Chemotherapy | 18F-AIF-NOTA-FAPI-04 | SUVmax, SUVmean, MTV, TLFAPI | RECIST 1.1 | FAPI PET/CT predicts survival and treatment response; ΔMTV more sensitive than ΔCA19-9. |
| Elboga et al. (13) | Turkey | retrospective | breast | 48 | 53 | all F | Chemotherapy | 68Ga-FAPI-04 | SUVmax, TBR | PERCIST | FAPI superior to FDG in accuracy; potential for complementary use in early chemotherapy response assessment. |
| Eshet et al. (14) | Israel | prospective | Invasive lobular breast cancer | 6 | 53 | all F | Hormonal therapy, chemotherapy, antibody-drug conjugates | 68Ga-FAPI-04 | SUVmax, tumor volume | RECIST | 68Ga-FAPI PET correlates with disease progression/regression in ILC and may be useful for response assessment. |
| Li et al. (15) | China | prospective | NHL | 18 | 56 | 10 M, 8 F | CHOP-like chemotherapy (R-CHOP, CHOP, R-COP) | 68Ga-FAPI-04 | SUVmax, SUVmean, SUVpeak, MTV, TLF, TBR | PERCIST 1.0 | Low FAPI uptake predicts poor response; SUVmax useful for screening |
| Miao et al. (16) | China | prospective | esophageal, gastric, colorectal | 17 | 61 | 11 M, 6 F | Surgery and Chemotherapy | 68Ga-FAPI-04 | SUVmax | NS | NS |
| Li et al. (17) | China | retrospective | colorectal | 13 | 48 | 7 F, 6 M | Combined TGF-β receptor inhibition and immunotherapy | 68Ga-FAPI-04 | SUVmax, TBR | NS | FAPI PET: noninvasive tool to assess tumor immunity and predict response to immunotherapy. Dual FAPI and FDG: recommended for colorectal management. |
| Cui et al. (18) | China | retrospective | ESCC, locally advanced | 19 | 58 | 18 M, 1 F | Neoadjuvant Chemoradiotherapy | 18F-NOTA-FAPI-04 | SUVmax, SUVmean, SUVpeak, MTV, TLFAPI | NS | Pretreatment MTV from FAPI PET/CT combined with platelet count can noninvasively predict response after treatment in ESCC. |
| Hua et al. (19) | China | prospective | glioma, recurrent | 30 | 51 | 20 M, 10 F | Prior standard treatment (surgery + radiotherapy/chemotherapy) | 68Ga-FAPI-04 | SUVmax, TBRmax, MTV, TLU | Visual assessment (PR, SD, PD) | MTV-FAPI is an independent risk factor for OS in recurrent glioma. |
| Zhang et al. (20) | China | retrospective | GI (pancreatic, cholangiocarcinoma, gastric, colorectal), peritoneal metastatic | 39 | 62 | 23 M, 16 F | Chemotherapy (gemcitabine-based, 5-FU-based, oxaliplatin-based, combination) | 18F-FAPI-04 | SUVmax, TBR | RECIST 1.1 | FAPI PET was effective in predicting the short-term outcomes of systemic chemotherapy for patients with peritoneal metastasis |
| Gao et al. (21) | Singapore | prospective | RCC, Primary and metastatic | 19 | NS | NS | NS | [18F]AlF-LNC1007 (FAPI-RGD); [18F]AlF-NOTA-FAPI-04 | SUVmax, SUVmean, TBR | IHC, Visual | NA |
| Lin et al. (22) | China | prospective | PDAC, Initial-stage (treatment-naive) | 67 | 63 | 45 M, 22 F | Initial-stage (treatment-naive) | F-FAPI-04 | SUVmax | Visual | NS |
| Michalski et al. (23) | Germany | retrospective | High-Grade Neuroendocrine Neoplasms (G3 NETs, NECs, MINENs), metastatic | 20 | 60 | 11 M, 9 F | Various (Chemotherapy, PRRT) | Ga-FAPI-04 | SUVmax, SUVmean, SUVpeak, Tumor Volume, Total Lesion Uptake | RECIST 1.1; Clinical signs of progression; Death | The presence of discordant FDG+/FAPI- lesions is associated with significantly shorter PFS, indicating more aggressive disease. |
| Sun et al. (24) | China | prospective | Gastric Adenocarcinoma (mucinous or signet ring cell component) | 49 | NS | NS | NS | Ga-FAPI | SUVmax | Visual assessment | NS |
| Zhang et al. (25) | China | prospective | Rectal, locally advanced | 20 | 60 | 13 M, 7F | Combined/modality therapy (Neoadjuvant Short-Course Radiotherapy + Immunochemotherapy) | Ga-FAPI-04 | SUVmax, SULpeak, FTV, TLF, TLR, Δ% changes | pCR | FAPI PET/MRI is promising for predicting response. ΔSULpeak decrease may guide selection of patients for non-surgical management. |
| Zhao et al. (26) | China | prospective | ESCC, locally advanced | 24 | NS | 21 M, 3 F | NS | F-FAPI-04 | SUVmax, SUVmean, SUVpeak, SUVsd, MTV, TLF, TBR | PD-L1 expression by IHC (22C3 assay) | FAPI PET/CT parameters correlate with PD-L1 expression in LA-ESCC. SUVsd was the optimal predictor. |
| Hu et al. (27) | China | prospective | ESCC, locally advanced | 18 | NS | 12 M, 6 F | Chemotherapy | F-FAPI-04 | SUVmax, SUVmean, SUVpeak, MTV, TLF, TBRblood, TBRmuscle | RECIST 1.1 | Baseline F-FAPI-04 TBRblood can predict short-term response to CCRT in LA-ESCC. |
| Miao et al. (28) | China | prospective | Gastric Adenocarcinoma, locally advanced | 28 | 61 | 21 M, 7 F | NAC | Ga-GAPI-04 | SUVmax, SUVpeak, TBR, %ΔSUVmax, %ΔSUVpeak, %ΔTBR | Tumor Regression Grade (pathology) | FAPI PET change rate parameters are superior to FDG for early prediction of pathologic response to treatment in LAGC. |
| Arak et al. (29) | Turkey | retrospective | Various Cancers (Breast, Lung, GI, Urogenital, Thyroid, etc.), Stage IV (with bone metastases) | 75 | 55 | 29 M, 46 F | NS | Ga-GAPI-04 | SUVmax | TPST 1.1 | FAPI PET SUVmax value is an independent prognostic factor for OS. |
| Watanabe et al. (30) | Germany | prospective | Various solid tumors (pancreatic, breast, thoracic, etc.), advanced (metastatic) | 145 | 62 | 85 M, 60 F | Various (chemotherapy, immunotherapy, etc.) | Ga-FAPI-46 | SUVmax | Visual assessment | Disease extent on FAPI PET/CT (total lesion number) is a predictor of short OS. |
| Rong et al. (31) | China | retrospective | gastric, metastatic | 21 | NS | NS | Immunotherapy | Ga-FAPI-04 | SUVmax, SUVmean | RECIST 1.1 | FAPI PET/CT can noninvasively predict response to anti-PD-1 therapy. |
| Zhang et al. (32) | China | retrospective | PDAC, Stage III or IV | 51 | 59 | 34 M, 17 F | NAC | Ga-DOTA-FAPI-04 | SUVmax, SUVmean, MTV, TLFAPI, ADC | RECIST 1.1 | Multiparameter [68Ga]Ga-DOTA-FAPI-04 PET/MR biomarkers (especially TLFAPI) are independent prognostic factors for PFS and OS. |
| Zheng et al. (33) | China | retrospective | breast | 64 | 48 | All F | NAC | Ga-FAPI-04 | SUVmax, SUVmean, FTV (40% SUVmax threshold), TLFAPI. Percentage changes calculated. | pCR | Serial FAPI-PET/CT parameters can predict response to therapy in breast cancer early (after 2 cycles) and pre-surgery. |
| Jiang et al. (34) | China | prospective | oral SCC, locally advanced (Stage III–IVA) | 20 | 47 | 18 M, 2 F | Neoadjuvant chemoimmunotherapy | Ga-FAPI-04 | SUVmax, SUVmean, SUVpeak. Percentage changes calculated. | pCR | ΔSUVs on FAPI-PET/CT are promising non-invasive biomarkers for predicting pathological response. |
| Qin et al. (35) | China | retrospective | gastric, All stages (I-IV) | 86 | 59 | 57 M, 29 F | Mixed: 40 had radical surgery (10 with neoadjuvant chemo); others had platinum/fluoropyrimidine chemotherapy. | Ga-FAPI-04 | SUVmax, SUVmean, FTV, TLF, TBR | RECIIST 1.1 | FAPI-PET heterogeneity and MTV metrics are independent prognostic factors for OS and PFS (in M0 patients). |
| Xu et al. (36) | China | prospective | HCC | 29 | 59 | NS | surgery | Ga-FAPI-04 | SUVmax, TBR | pCR | FAPI and [11C]-acetate have good predictive value in HCC. Their combination has the highest sensitivity for detection. |
| Beyhan et al. (37) | Turkey | prospective | gastric | 31 | 59 | 23 M, 8 F | NS | Ga-FAPI-04 | SUVmax, SUVmean, TBR | Histopathology, MRI, follow-up | NS |
| Zhang et al. (38) | China | prospective | PDAC, locally advanced | 34 | 65 | 21 M, 13 F | NAC | [18F]AlF-NOTA-FAPI-04 | SUVmax, MTV, TLFAPI, FPV, TPF | Evans Tumor Regression Grade (pathology) | FAPI PET/CT parameters can predict pathological response to NAC and are independent prognostic factors for survival. |
| Ding et al. (39) | China | prospective | PDAC, Localized (potentially resectable) on CT (I-IV) | 30 | 61 | 26 M, 23 F | Surgery, Neoadjuvant Chemo, Systemic Chemo | Ga-FAPI-04 | SUVmax, MTV, TLFAPI | Pathology, follow-up | FAPI uptake correlates with aggressive pathology and provides significant prognostic value. |
| Ding et al. (40) | China | prospective | PDAC, resectable / borderline resectable (AJCC I-III) | 37 | 61 | 23 M, 14 F | Surgery followed by adjuvant chemo (Tegafur, Gemcitabine, FOLFIRINOX, etc.), neoadjuvant (imaging pre-surgery) | Ga-FAPI-04 | SUVmax, MTV, TLFAPI | pCR | NS |
| Zhao et al. (41) | China | prospective | ESCC, locally advanced (T2-T4, N0-N3, M0; AJCC II-IVA) | 34 | 65 | 26 M, 8 F | Definitive Radiochemotherapy | Ga-FAPI-04 | SUVmax, SUVmean, GTV, TLFAPI | RECIST 1.1 | MTV is an independent prognostic factor for PFS and OS in patients treated with radiochemotherapy. |
| Zhang et al. (42) | China | retrospective | Pancreatic cancer, borderline resectable or locally advanced | 18 | 55 | 13 M, 5 F | NAC | Ga-FAPI-04 | SUVmax, TLFAPI, TBR, SUVmean, MTV SUVpeak, | RECIST 1.1 | Baseline FAPI PET/MR parameters can predict response after therapy. |
| Chen et al. (43) | China | prospective | Breast, stages IIA-IIIC | 22 | NS | All F | NAC, surgery | Ga-FAPI | SUVmax, TBR, ΔSUVmax | pCR | Assessment of early changes in FAPI uptake can predict pCR. FAPI PET has great potential for early and late prediction of pathologic response to NAC. |
| Metzger et al. (44) | Germany | retrospective | PDAC, locally advanced/recurrent | 27 | 61 | 15 F, 12 M | CRT, Consolidation after chemotherapy | Ga-FAPI-46 | SUVmax, SUVmean, FAPI-derived tumor volume, TLFAPI | Evaluated with previous CT, MRI, EUS, or histopathology | Decreases in SUVmax/SUVmean post-CRT might be associated with favorable local tumor control. |
| Wu et al. (45) | China | prospective | Unresectable HCC | 22 | 62 | 19 M, 3 F | Combination (PD-1 Inhibitor + Lenvatinib), First-line or later | Ga-FAPI-04 | SUVmax, FAPI-avid tumor volume, TLFAPI | Modified RECIST | Volumetric indices on baseline FAPI are potential independent prognostic factors for predicting benefit and survival from therapy. |
| Jia et al. (46) | China | prospective | PDAC, locally advanced or metastatic | 28 | 57.5 | 19 M, 9 F | Combined Immunotherapy + Chemotherapy or Chemotherapy alone | Ga-FAPI-04 | SUVmax, SUVmean, TBR, MTV. | RECIST 1.1 | Baseline TBR on FAPI PET predicts poor response. |
| Selcuk et al. (47) | Turkey | retrospective | GI (Gastric, Colorectal, Pancreatic, etc.), primary, recurrent and metastatic. | 50 | 61.62 | 24 M, 26 F | Chemotherapy, Tyrosine Kinase Inhibitors. | Ga-DOTA-FAP-2286 | SUVmax, Total Tumor Volume, TLFAPI | PERCIST (for FAPI-PET/CT) and RECIST 1.1 (for CT) | FAPI PET/CT is effective for treatment response assessment and predicts overall survival. |
| PDAC: Pancreatic Adenocarcinoma, GIST: Gastrointestinal Stromal Tumor, NHL: Non-Hodgkin Lymphoma, ESCC: Esophageal Squamous Cell Carcinoma, HCC: Hepatocellular Carcinoma, F: Female, M: Male, NAC: Neoadjuvant Chemotherapy, CRT: Chemoradiotherapy, SUV: Standardized Uptake Value, TBR: Tumor-Background Ratio, MTV: FAPI-avid Tumor Volume, TLFAPI: Total Lesion FAP Expression, PR: Partial Response, SD: Stable Disease, PD: Progressive Disease, pCR: pathologic Complete Response, | | | | | | | | | | | |

**References**

1.	Backhaus P, Burg MC, Asmus I, Pixberg M, Büther F, Breyholz H-J, et al. Initial results of 68Ga-FAPI-46 PET/MRI to assess response to neoadjuvant chemotherapy in breast cancer. Journal of Nuclear Medicine. 2023;64(5):717-23.

2.	Çermik TF, Ergül N, Yılmaz B, Mercanoğlu G. Tumor imaging with 68Ga-DOTA-FAPI-04 PET/CT: comparison with 18F-FDG PET/CT in 22 different cancer types. Clinical nuclear medicine. 2022;47(4):e333-e9.

3.	Helisch A, Kratochwil C, Kleist C, Krämer S, Castillo JJR, Dendl K, et al. Feasibility, tolerability, and preliminary clinical response of fractionated radiopharmaceutical therapy with 213Bi-FAPI-46: pilot experience in patients with end-stage, progressive metastatic tumors. Journal of Nuclear Medicine. 2024;65(12):1917-22.

4.	Dong Y, Wang Z, Hu X, Sun Y, Qin J, Qin Q, et al. [18F] AlF-NOTA-FAPI-04 PET/CT for Predicting Pathologic Response of Resectable Esophageal Squamous Cell Carcinoma to Neoadjuvant Camrelizumab and Chemotherapy: A Phase II Clinical Trial. Journal of Nuclear Medicine. 2024;65(11):1702-9.

5.	Chen R, Yang X, Ng YL, Yu X, Huo Y, Xiao X, et al. First total-body kinetic modeling and parametric imaging of dynamic 68Ga-FAPI-04 PET in pancreatic and gastric cancer. Journal of Nuclear Medicine. 2023;64(6):960-7.

6.	Wu C, Wen F, Lin F, Zeng Y, Lin X, Hu X, et al. Predictive performance of [18F] F-fibroblast activation protein inhibitor (FAPI)-42 positron emission tomography/computed tomography (PET/CT) in evaluating response of recurrent or metastatic gastrointestinal stromal tumors: complementary or alternative to [18F] fluorodeoxyglucose (FDG) PET/CT? Quantitative imaging in medicine and surgery. 2024;14(8):5333.

7.	Novruzov E, Giesel FL, Mori Y, Choyke PL, Dabir M, Mamlins E, et al. Head-to-head intra-individual comparison of biodistribution and tumor uptake of [18F] FAPI-74 with [18F] FDG in patients with PDAC: a prospective exploratory study. Cancers. 2023;15(10):2798.

8.	Xiong M, You H, Feng J, Liu Y, Luo X, Liu Y, et al. 18F-FAPI-42 PET/CT and 18F-FDG PET/CT in patients with malignant digestive system neoplasms: a head-to-head comparative study. Molecular Imaging and Biology. 2025;27(1):131-41.

9.	Li X, Lu N, Lin L, Chen Y, Yang S, Wang H, et al. 18F-FAPI-04 outperforms 18F-FDG PET/CT in clinical assessments of patients with pancreatic adenocarcinoma. Journal of Nuclear Medicine. 2024;65(2):206-12.

10.	Chen R, Yang X, Yu X, Zhou X, Ng YL, Zhao H, et al. Tumor-to-blood ratio for assessment of fibroblast activation protein receptor density in pancreatic cancer using [68Ga] Ga-FAPI-04. European Journal of Nuclear Medicine and Molecular Imaging. 2023;50(3):929-36.

11.	Li X, Lu N, Sun K, Shi F, Lin L, Chen Y, et al. [18F] FAPI-04 PET/CT for pathologic response assessment in pancreatic cancer patients with systemic treatment. European Journal of Nuclear Medicine and Molecular Imaging. 2025:1-13.

12.	Zhu Z, Cheng K, Yun Z, Zhang X, Hu X, Liu J, et al. [18F] AlF-NOTA-FAPI-04 PET/CT can predict treatment response and survival in patients receiving chemotherapy for inoperable pancreatic ductal adenocarcinoma. European Journal of Nuclear Medicine and Molecular Imaging. 2023;50(11):3425-38.

13.	Elboga U, Sahin E, Kus T, Cayirli YB, Aktas G, Uzun E, et al. Superiority of 68Ga-FAPI PET/CT scan in detecting additional lesions compared to 18FDG PET/CT scan in breast cancer. Annals of Nuclear Medicine. 2021;35(12):1321-31.

14.	Eshet Y, Tau N, Levanon K, Bernstein-Molho R, Globus O, Itay A, et al. The role of 68Ga-FAPI PET/CT in breast cancer response assessment and follow-up. Clinical Nuclear Medicine. 2023;48(8):685-8.

15.	Li L, Ding H, Chen L, Peng D, Chen Y. Metabolic response prediction using 68Ga-FAPI PET/CT in Non-Hodgkin lymphoma treated with chemotherapy: a pilot study. Cancer Imaging. 2025;25(1):69.

16.	Miao J, Xu Y, Zhao X, Su X, Zhu G, Wu T, et al. Atypical Appearance of Recurrence and Metastasis of Gastrointestinal Carcinomas and Its Treatment Response: Solve 18F-FDG PET/CT Challenging Cases Using 68Ga-FAPI PET/MRI: a case-control study. 2024.

17.	Li K, Liu W, Yu H, Chen J, Tang W, Wang J, et al. 68 Ga-FAPI PET imaging monitors response to combined TGF-βR inhibition and immunotherapy in metastatic colorectal cancer. The Journal of clinical investigation. 2024;134(4).

18.	Cui Y, Wang H, Wang N, Yao Z, Li Z, Wu Q, et al. 18F-NOTA-FAPI-04 PET/CT metabolic parameters combined with hematological biomarkers predict pathological complete response after neoadjuvant chemoradiotherapy in esophageal squamous cell carcinoma. European Journal of Nuclear Medicine and Molecular Imaging. 2025:1-10.

19.	Hua T, Huang Q, Zhou Z, Zhou W, Wen J, Xie F, et al. Fibroblast activation protein expression in the tumor microenvironment is crucial in survival prediction and differentiation of recurrent gliomas: a head-to-head comparison of 68Ga-FAPI-04 and 18F-FET in PET/CT imaging. EJNMMI Radiopharmacy and Chemistry. 2025;10(1):57.

20.	Zhang Y, Xu M, Wang Y, Yu F, Chen X, Wang G, et al. Characteristics of 18F-FAPI-04 PET/CT in patients with peritoneal metastasis and to predict treatment efficacy, a head-to-head comparison with 18F-FDG PET/CT. Cancer Imaging. 2025;25(1):66.

21.	Gao H, Ma Z, Zhu Z, Yang Z, Chen B, Wu X, et al. Comparative study of [18F] AlF-NOTA-FAPI-RGD and [18F] FDG/[18F] AlF-NOTA-FAPI-04 PET/CT in renal cell carcinoma. Theranostics. 2025;15(12):5790.

22.	Lin L, Wang G, Zhang Y, Wang G, Zhao K, Su X. Clinical Utility of 18Fluorine‐Fibroblast Activation Protein Inhibitor‐04 Positron Emission Tomography/Computed Tomography in the Evaluation of Pancreatic Ductal Adenocarcinoma: Comparison With 18Fluorine‐Fluorodeoxyglucose Positron Emission Tomography/Computed Tomography. MedComm. 2025;6(3):e70136.

23.	Michalski K, Kosmala A, Hartrampf PE, Heinrich M, Serfling SE, Schlötelburg W, et al. [18F] FDG and [68Ga] Ga-FAPI-04–Directed Imaging for Outcome Prediction in Patients with High-Grade Neuroendocrine Neoplasms. Journal of Nuclear Medicine. 2024;65(12):1899-903.

24.	Sun Y, Xu J, Qiao Y, Zhang J, Pan H, Xu X, et al. Assessing the value of 68Ga-FAPI PET/CT in gastric mucinous adenocarcinoma or signet ring cell carcinoma. Radiology: Imaging Cancer. 2024;6(6):e230195.

25.	Zhang X, Lin Z, Feng Y, Lin Z, Tao K, Zhang T, et al. Predicting pathologic complete response in locally advanced rectal cancer with [68Ga] Ga-FAPI-04 PET,[18F] FDG PET, and contrast-enhanced MRI: lesion-to-lesion comparison with pathology. Journal of Nuclear Medicine. 2024;65(10):1548-56.

26.	Zhao Y, Ren J. 18F-FAPI-04 PET/CT parameters predict PD-L1 expression in esophageal squamous cell carcinoma. Frontiers in Immunology. 2023;14:1266843.

27.	Hu X, Zhou T, Ren J, Duan J, Wu H, Liu X, et al. Response prediction using 18F-FAPI-04 PET/CT in patients with esophageal squamous cell carcinoma treated with concurrent chemoradiotherapy. Journal of Nuclear Medicine. 2023;64(4):625-31.

28.	Miao Y, Feng R, Yu T, Guo R, Zhang M, Wang Y, et al. Value of 68Ga-FAPI-04 and 18F-FDG PET/CT in early prediction of pathologic response to neoadjuvant chemotherapy in locally advanced gastric cancer. Journal of Nuclear Medicine. 2024;65(2):213-20.

29.	Arak H, Elboga U, Cayirli YB, Aytekin A. Prognostic significance of 68 Ga-FAPI PET/CT in patients with bone metastases in various cancers. Annals of Nuclear Medicine. 2024;38(8):630-8.

30.	Watanabe M, Fendler WP, Grafe H, Hirmas N, Hamacher R, Lanzafame H, et al. Prognostic Implications of 68Ga-FAPI-46 PET/CT–Derived Parameters on Overall Survival in Various Types of Solid Tumors. Journal of Nuclear Medicine. 2024;65(7):1027-34.

31.	Rong X, Lv J, Liu Y, Wang Z, Zeng D, Li Y, et al. PET/CT imaging of activated cancer-associated fibroblasts predict response to PD-1 blockade in gastric cancer patients. Frontiers in Oncology. 2022;11:802257.

32.	Zhang Z, Guo S, Su W, Pan G, Wang Y, Li Y, et al. Prognostic value of multiparameter [68Ga] Ga-DOTA-FAPI-04 PET/MR imaging biomarkers for patients with advanced pancreatic cancer. European Journal of Nuclear Medicine and Molecular Imaging. 2025:1-12.

33.	Zheng S, Xu F, Chen Y, Shao Z, Wu Z, Liu Q, et al. The value of serial [⁶⁸Ga] Ga-FAPI-04 PET/CT in predicting pathological response and evaluating therapeutic efficacy to neoadjuvant chemotherapy in breast cancer. European Journal of Nuclear Medicine and Molecular Imaging. 2025:1-12.

34.	Jiang Y, Yu Z, Sun Y, Tian Y, Wang L, Xing D, et al. Predictive value of [68Ga] Ga-FAPI-04 PET/CT on pathologic response to neoadjuvant chemoimmunotherapy for locally advanced resectable oral squamous cell carcinoma. European Journal of Nuclear Medicine and Molecular Imaging. 2025:1-12.

35.	Qin C, Fu Y, Zhang X, Li M, Ruan W, Gai Y, et al. Prognostic value of [68Ga] Ga-FAPI-04 PET in patients with newly diagnosed gastric carcinoma. European Journal of Nuclear Medicine and Molecular Imaging. 2025;52(9):3075-86.

36.	Xu H, Wang H, Yu S-z, Li X-m, Jiang D-l, Wu Y-f, et al. Prognostic and diagnostic value of [18F] FDG, 11C-acetate, and [68Ga] Ga-FAPI-04 PET/CT for hepatocellular carcinoma. European Radiology. 2025:1-11.

37.	Beyhan E, Çermik TF, Fenercioğlu ÖE, Şahin R, Alçin G, Aksoy T, et al. The contribution of 68Ga-FAPI-04 PET/CT to staging and prognosis in gastric cancer. Clinical Nuclear Medicine. 2024;49(10):e485-e91.

38.	Zhang Y, Xu M, Wang Y, Yu F, Chen X, Wang G, et al. Value of [18F] AlF-NOTA-FAPI-04 PET/CT for predicting pathological response and survival in patients with locally advanced pancreatic ductal adenocarcinoma receiving neoadjuvant chemotherapy. European Journal of Nuclear Medicine and Molecular Imaging. 2025;52(6):2118-31.

39.	Ding J, Qiu J, Hao Z, Huang H, Liu Q, Liu W, et al. Comparing the clinical value of baseline [68 Ga] Ga-FAPI-04 PET/CT and [18F] F-FDG PET/CT in pancreatic ductal adenocarcinoma: additional prognostic value of the distal pancreatitis. European Journal of Nuclear Medicine and Molecular Imaging. 2023;50(13):4036-50.

40.	Ding J, Qiu J, Hao Z, Huang H, Liu Q, Liu W, et al. Prognostic value of preoperative [68 Ga] Ga-FAPI-04 PET/CT in patients with resectable pancreatic ductal adenocarcinoma in correlation with immunohistological characteristics. European Journal of Nuclear Medicine and Molecular Imaging. 2023;50(6):1780-91.

41.	Zhao L, Pang Y, Chen S, Chen J, Li Y, Yu Y, et al. Prognostic value of fibroblast activation protein expressing tumor volume calculated from [68 Ga] Ga-FAPI PET/CT in patients with esophageal squamous cell carcinoma. European Journal of Nuclear Medicine and Molecular Imaging. 2023;50(2):593-601.

42.	Zhang Z, Guo S, Cheng C, Cao K, Jiang H, Jin G, et al. Integrated 68Ga-FAPI-04 PET/MR in pancreatic cancer: prediction of tumor response and tumor resectability after neoadjuvant therapy. Clinical Nuclear Medicine. 2024;49(8):715-21.

43.	Chen L, Zheng S, Chen L, Xu S, Wu K, Kong L, et al. 68Ga-labeled fibroblast activation protein inhibitor PET/CT for the early and late prediction of pathologic response to neoadjuvant chemotherapy in breast cancer patients: a prospective study. Journal of Nuclear Medicine. 2023;64(12):1899-905.

44.	Metzger G, Bayerl C, Rogasch JM, Furth C, Wetz C, Beck M, et al. 68Ga-labeled fibroblast activation protein inhibitor (FAPI) PET/CT for locally advanced or recurrent pancreatic cancer staging and restaging after chemoradiotherapy. Theranostics. 2024;14(11):4184.

45.	Wu M, Wang Y, Yang Q, Wang X, Yang X, Xing H, et al. Comparison of baseline 68Ga-FAPI and 18F-FDG PET/CT for prediction of response and clinical outcome in patients with unresectable hepatocellular carcinoma treated with PD-1 inhibitor and lenvatinib. Journal of Nuclear Medicine. 2023;64(10):1532-9.

46.	Jia R, Hao Z-X, Zhang N, Si H-Y, Zhu W-J, Wang Z-K, et al. Predictive Value of 68Ga‑FAPI PET/CT in Patients Receiving First-Line Therapy for Pancreatic Ductal Adenocarcinoma. CT in Patients Receiving First-Line Therapy for Pancreatic Ductal Adenocarcinoma.

47.	Selcuk NA, Beydagi G, Akcay K, Gormez A, Toklu T, Kabasakal L. The role of [⁶⁸Ga] Ga-FAP-2286 PET/CT in the evaluation of treatment response in gastrointestinal system malignancies. European Journal of Nuclear Medicine and Molecular Imaging. 2025:1-7.
