# Supplementary material for: Predicting the unpredictable: Prognostic and predictive power of FAPI-PET imaging in oncology: A systematic review and meta-analysis
Source: Eur J Nucl Med Mol Imaging. 2026 Jan 27;53(5):2927–38. doi: 10.1007/s00259-025-07735-9 (PMC13649892; doi:10.1007/s00259-025-07735-9)
Supplement: Supplementary file 2 [file 259_2025_7735_MOESM2_ESM.docx]

***Supplementary Table 2. Risk-of-bias of the included studies using the Oxford University for CEBM checklist for Prognostic Studies.***

| Author | Representativeness of cohort | Follow-up | Outcome measurement | Adjustment for confounding | Overall |
| --- | --- | --- | --- | --- | --- |
| Backhaus et al. (1) | Small sample, single-center, retrospective; likely selection bias. | Short-term outcome post-therapy; no lost follow-up. | Treatment response defined by histopathology (gold standard); blinded readers for imaging. | No multivariate analysis; small sample limits adjustment. | Small sample, retrospective, lack of adjustment for confounders. |
| Cermik et al. (2) | Only one patient for treatment response evaluation. | Not reporting any longitudinal follow-up data for outcomes. The outcome being reported is diagnostic performance, not prognosis. | For its stated aim (diagnostic comparison), the PET/CT images were interpreted by experienced specialists. | No adjustment for confounding factors like cancer type, stage, patient age, or treatment received. | Lacking the fundamental components of a prognostic study: a well-defined inception cohort, longitudinal follow-up, and correlation of the FAPI uptake with hard clinical endpoints. |
| Helisch et al. (3) | small sample, end-stage, heavily pretreated; heterogeneous cancers; not representative. | Short follow-up; no long-term outcomes. | PET-based (TLFAPI) and visual assessment; not blinded. | No adjustment; small number, heterogeneous group. | Small sample, descriptive analysis, heterogeneous population, short follow-up. |
| Dong et al. (4) | n=32, single-arm phase II trial; well-defined inclusion criteria. | Pathologic response assessed immediately post-surgery. | Tumor Regression Grade by Chirieac system (standardized pathology); blinded imaging assessment. | Logistic regression not performed; univariate analysis only. | Single-center, modest sample size, no multivariate adjustment. |
| Chen et al. (5) | Various GI cancers; specific disease stage and prior treatment history are not detailed in a way that defines a common inception point. | Not reporting any patient follow-up data or correlate the derived kinetic parameters (like V_t_) with survival or treatment response. | The outcome measures are technical (SUV, V_t_, SNR, CNR). There is no mention of how clinical outcomes were assessed, as they were not the focus of the study. | No adjustment for confounding factors was performed, as the study did not aim to establish a prognostic model. | Primary aim of validating kinetic modeling and parametric imaging for FAPI. Not providing data on the relationship between FAPI-derived kinetic parameters and patient outcomes. |
| Wu et al. (6) | Included patients at a specific point: before switching targeted therapy. This is not a common, early point in the disease course but rather a later, treatment-refractory stage. The spectrum of patients is narrow and highly selected. | The follow-up for the primary outcome (treatment response based on RECIST) was standardized and likely sufficient for that endpoint. | Objective, based on RECIST. PET parameters: tested as predictive factors, measured by the blinded researchers. Outcome assessment on CT was likely done separately, non-blinded measurement of key prognostic factor. | Developed a multivariate model that integrated PET parameters with established clinical prognostic factors. | A predictive, not a pure prognostic, study. The narrow, late-stage inception cohort and the lack of blinding in factor measurement contribute to high risk of bias for general prognostic conclusions. |
| Novruzov et al. (7) | Include only one patient for therapy response control after neoadjuvant therapy. | Not providing follow-up data to see if FAPI-positive lesions indeed represented progressive disease or if the FAPI findings predicted subsequent survival or treatment failure. | Lesion detection: based on imaging and follow-up, but the primary outcome was diagnostic performance, not a clinical outcome. The assessment was not blinded to the clinical context in a way that would be required for a robust prognostic study. | With only 7 patients and a focus on intra-individual comparison of tracer uptake, no multivariate analysis was possible to adjust for other factors influencing prognosis. | Not linking the quantitative FAPI metrics (SUVmax) to future patient outcomes. It demonstrates the potential for FAPI to be prognostic, but it does not directly test or prove a prognostic hypothesis. |
| Xiong et al. (8) | A mixed group of patients at different stages of GI cancers. A heterogeneous cohort not assembled at a common, early point in the disease. The inclusion of an "additional FAPI-PET" arm based on physician discretion introduces significant selection bias. | Diagnostic focus of primary outcome (sensitivity, specificity); not investigating the association between FAPI uptake and survival outcomes. | Reference standard: a combination of pathology, typical imaging, and clinical follow-up. The visual and quantitative analysis of PET scans was performed by two non-blinded physicians. The objectivity of the primary outcomes is limited. | Descriptive and comparative between two imaging modalities. No adjustment of FAPI uptake for other clinical variables to determine its independent prognostic value. | A diagnostic accuracy, not a prognostic study. Not meeting the fundamental criteria for assessing prognosis, as it lacks both a standardized inception cohort and any analysis of survival outcomes. |
| Li et al. (9) | Specific to pancreatic adenocarcinoma. | Study reports on a predictive outcome (treatment response by RECIST) but does not provide data on survival outcomes (PFS/OS). | RECIST as an objective assessment. The PET/CT analysis was performed by non-blinded physicians, potentially introducing bias in the quantitative measurements. | Univariate analysis of the predictive value for treatment response. Not performing multivariate analysis to adjust FAPI uptake for other known prognostic factors (e.g., CA19-9, stage). | A diagnostic accuracy, not a prognostic study. Not meeting the fundamental criteria for assessing prognosis, lacking both standardized inception cohort and any analysis of survival outcomes. |
| Chen et al. (10) | A small, highly selected sample, making it unlikely to be representative of the broader pancreatic cancer population. | Focused on correlating TBR with Vt and assessing early changes post-therapy; standard follow-up criteria do not apply. | The primary outcomes of this study were the technical parameters Vt and TBR/SUV. These are quantitative, objective measurements. The correlation between TBR and Vt is not susceptible to observer bias. | No adjustment for any other prognostic factors (e.g., stage, performance status, CA19-9). The study was not designed for this purpose. | High risk of bias in representativeness and adjustment. Its value lies in providing a physiological rationale for using TBR as a surrogate for FAP receptor density. |
| Li et al. (11) | Critical prognostic and predictive analysis: performed on a highly selected subgroup, representing a minority of pancreatic cancer patients with favorable biology and response to therapy, not representative of the general population. Small sample size. | Explicitly stated to be short. The authors note that the lack of significant survival differences may be due to this short follow-up. | The use of pathologic treatment response based on CAP score is the gold standard and highly objective. The PET parameters are quantitative. This is a major strength of the study. | The survival analysis was performed only based on the median values of ΔTBR metrics. There was no adjustment for any other prognostic factors in a multivariate model. | High-quality evidence for the predictive value of FAPI for pathological response. Highly selected cohort, short follow-up, and lack of adjustment for confounders in survival analysis. |
| Zhu et al. (12) | Prospective, single center. focused on "inoperable PDAC," making the sample representative of that specific subpopulation. | The study clearly defines OS and PFS and uses standard statistical methods; implies a reasonably complete follow-up for the cohort. | The primary prognostic outcome was objective. The imaging parameters are quantitative. For the predictive analysis of treatment response, RECIST was used, which is a standard, albeit imaging-based, criterion. | Performing both univariate and multivariate Cox regression analyses. It adjusts for clinical covariates for OS. For PFS, the adjustment appears less comprehensive. | Clear prospective design, defined patient population, objective outcomes, and attempting to adjust for key confounders. Main limitations are the single-center design and the relatively small sample for subgroup analyses. |
| Elboga et al. (13) | Retrospective, included 48 breast cancer patients from a single institution. The inclusion of patients with "locally advanced or metastatic stage disease" suggests a selected, more severe patient population. | A diagnostic accuracy study, not a longitudinal prognostic study. The primary outcome was the detection of lesions and its impact on immediate treatment management, not long-term survival outcomes. Therefore, formal "follow-up" for survival was not a stated aim. | "Change in treatment response assessment”; based on PERCIST criteria, which are semi-objective. However, the reclassification of 12 patients was based on the new scan findings without blinding to the clinical context, introducing potential for bias. | Did not aim to establish FAPI as a prognostic factor. It was a descriptive comparison of two imaging modalities. No multivariate analysis was performed to adjust for known prognostic factors like cancer subtype, stage, or treatment history. | It lacks a representative inception cohort, has no long-term follow-up for survival, and makes no attempt to adjust for confounding factors. Its value for a prognostic meta-analysis is limited to illustrating how FAPI can reclassify patients into different response categories, a potential surrogate for prognosis. |
| Eshet et al. (14) | The study enrolled a very small, highly selected cohort from a previous study, all with FAPI-avid metastatic ILC. not representative of the broader breast cancer or even ILC population. | The follow-up appears complete for the included participants. No loss to follow-up is reported. | Not a hard endpoint like OS or PFS, but blood tumor biomarkers and qualitative CT assessment. Biomarkers can be unreliable, and CT is known to be insensitive for ILC, making them imperfect reference standards. | Not accounting for important prognostic confounders such as type of therapy, sites of metastasis. The small sample size makes any adjustment impossible. | Small, selected sample, the use of surrogate outcomes, and potential statistical issues in the analysis. Its primary contribution is generating hypothesis. |
| Li et al. (15) | Consecutive enrollment, small number. The heterogeneity of NHL subtypes (DLBCL, MCL, etc.) treated with slightly different regimens (CHOP vs. R-CHOP) limit the generalizability and representativeness. | All enrolled participants completed the baseline and follow-up scans and were included in the analysis. | The outcome (metabolic response) was assessed using FDG PET/CT and PERCIST 1.0 criteria, which is a standard and objective methodology in oncology. | The study measured some confounders but due to the small number of events, the multivariate analysis was limited. It is unlikely that all important confounders were adequately accounted for. | Small, heterogeneous sample and the inability to fully account for confounding. Findings require validation in larger, subtype-specific cohorts. |
| Miao et al. (16) | A prospective but small (n=22) single-center study. It specifically selected patients where FDG was challenging (e.g., post-treatment, low-metabolism tumors). A highly selected, niche population. | Primarily a diagnostic accuracy study. The outcomes are detection rates and semi-quantitative uptake values (SUVmax). While "follow-up" was used as a reference standard for some lesions, the study does not report longitudinal survival data or treatment response over time. | The primary outcome was diagnostic performance (sensitivity, specificity). The reference standard was a composite of pathology, follow-up imaging, and endoscopy. While objective, the lack of a uniform, blinded reference for all lesions is a minor limitation. | The study did not perform any analysis to determine if FAPI uptake was an independent predictor of outcomes. It was purely focused on diagnostic superiority in complex cases. | Providing excellent evidence for the diagnostic utility of FAPI PET/MRI in specific, challenging clinical scenarios in GI cancers. However, it does not address prognostic value or predictive value for long-term treatment response, as it lacks the necessary longitudinal design and statistical analysis. |
| Li et al. (17) | Large cohort for a PET imaging study and includes a well-defined population. The inclusion of patients who received immunotherapy provides a relevant sub-cohort for predictive analysis. | For the immunotherapy subgroup, outcomes are reported for all. | The outcome in the immunotherapy subgroup is clinical response. The assessment is not blinded and is based on standard clinical/radiological follow-up, which can introduce bias. | In clinical immunotherapy subgroup, no adjustment for confounders was possible; small sample size. The strong association found may be influenced by confounding. | Small, unadjusted subgroup analysis. Only providing powerful hypothesis-generating tool for future predictive studies. |
| Cui et al. (18) | Retrospective. Includes only patients who were selected for and completed NAC followed by surgery. The cohort is therefore not an inception cohort but a selected subgroup of patients with a specific treatment pathway. | The primary outcome was pCR, assessed immediately after surgery. Therefore, attrition is not a concern for this outcome. | The outcome was determined by histopathological examination of the resected specimen, which is the gold standard. This was performed by senior pathologists using a double-blind protocol, minimizing assessment bias. | Despite doing the multivariate logistic regression for the MTV, it was not performed for other parameters. | Non-inception cohort design, and mall sample size; increases the risk of overfitting the statistical model and limits the generalizability. Lack of survival data restricts its prognostic value. |
| Hua et al. (19) | Enrolled patients at a point of suspected recurrence, not at the initial diagnosis or the start of a standard treatment. This is a "trigger-based" cohort, which is susceptible to work-up bias. Spectrum of disease at this stage is variable. | Not explicitly reporting the completeness of follow-up (were all patients followed until death or a censoring date?). Small sample size; small amount of attrition could significantly bias the results. | Reference standard: combination of histopathology and "authorized diagnoses" from expert consultations for others. This introduces potential incorporation bias, as the PET results or clinical data may have influenced the "authorized diagnosis," and it is not uniform gold standard for all patients. | Multivariate logistic regression was performed for confounding factors. | Non-inception cohort design, the use of a non-uniform reference standard for the differentiation outcome, and the unclear completeness of survival follow-up contribute to high risk of bias. The small sample size is also a concern. |
| Zhang et al. (20) | Assembling patients with established and pathologically confirmed metastases who are about to start chemotherapy; not an early point in the disease course but rather a late, specific stage. | Primary outcome assessed by RECIST. All patients were included in this analysis; no attrition for this outcome. | Treatment response was assessed using RECIST criteria on contrast-enhanced CT; an objective and standard oncological endpoint. The assessments were made by specialists blinded to the PET results. | No adjustment was done. | Well-defined patient group, head-to-head comparison with FDG-PET, clear and objectively measured primary outcome, and robust correlation with histology. |
| Gao et al. (21) | Prospective, single-center, recruiting 35 patients "highly suspected to have RCC." The cohort is relatively. While prospective, it may not be fully representative of the entire spectrum of RCC patients. | Follow-up of 26 months to confirm lesions without pathology. However, the primary outcomes are diagnostic efficacy. The follow-up was used for lesion validation, not for prognostic analysis. | Outcomes were well-defined: detection rates based on pathology/imaging follow-up, and semi-quantitative SUVmax/TBR measurements. Blinded imaging analysis and IHC scoring. | Aim: diagnostic, not prognostic. Not investigating the association between tracer uptake and survival or treatment response, performed no multivariate analysis to adjust for confounders. | A high-quality diagnostic study. Providing crucial data on the correlation between tracer uptake and underlying tumor biology. However, it does not bridge this finding to clinical outcomes like survival, which is the core of a prognostic study. |
| Lin et al. (22) | Single center. While the clinical characteristics are well-described, it is a selected cohort from one institution, which may not be fully representative of the broader PDAC population. | This study is primarily diagnostic and cross-sectional in its main analysis. It does not report on patient survival or long-term treatment response follow-up. | Objective outcomes of SUVmax. For the prediction of pathological features (like differentiation), the outcome (pathology) was objective but it's unclear if the PET readers were blinded to it. | The study performed univariate and multivariate analyses to identify risk factors for metastasis. Adjusting for other variables like ascites and CA19-9. | Not a longitudinal prognostic study. Its main focus is diagnostic performance and correlation with pathological features at baseline. While it identifies potential prognostic risk factors. |
| Michalski et al. (23) | Single-center, retrospective study of only 20 patients with highly aggressive NENs, a rare and heterogeneous group. | Appropriate PFS of 8 months for this aggressive disease. Not explicitly reporting the patients lost to follow-up or completeness of the follow-up data. PFS was defined using a combination of RECIST, clinical, and survival endpoints. | PFS was defined using standardized, pre-specified criteria. The PET readers were likely blinded to the eventual PFS outcomes at the time of scan interpretation. | No adjustment was done. | The major limitations are the very small, non-representative sample and the lack of multivariate adjustment for confounding factors. |
| Sun et al. (24) | Prospective, single-center, consecutive recruitment. The sample size was calculated a priori. | Following until progression or a defined period. The PFS of 9.4 months, and the overall disease-progression rate of 67.4%; sufficient follow-up duration. | PFS was defined as the time from the PET/CT scan to disease progression. Blinded readers of the PET/CT scans to clinical data and the results of the other scans. | Adjustment was done using multivariate logistic regression. | Well-designed prospective study with a clear methodology, blinded assessment, and appropriate statistical adjustment in a multivariable model. |
| Zhang et al. (25) | Prospective. Single-center, specific and uniform treatment. Small sample size, all patients had proficient mismatch repair/microsatellite-stable tumors. | All patients completed the trial and surgery, providing a definitive pathological outcome for all participants. There was no loss to follow-up for the primary endpoint. | Determined by histopathological examination of the resected surgical specimen, which is the gold standard and objective. The PET parameters were quantified independently of the pathological assessment. | No adjustment was done. | Prospective design, use of a gold-standard outcome. Small sample size, the lack of adjustment for other potential predictors in multivariable model. |
| Zhao et al. (26) | Prospective, single center, small sample size. | Cross-sectional; correlating baseline FAPI-PET parameters with baseline PD-L1 expression. There is no follow-up. | PD-L1 expression (by IHC); an objective pathological criterion. The PET parameters were also quantitative. Blinded pathologists and imaging physicians. | Used logistic regression. As the small sample size, the model is possibly overfitted. The confidence intervals for the odds ratios are wide, indicating imprecision. | Investigated a predictive biomarker correlation. The small sample size, particularly in the positive PD-L1 group, is a source of bias and imprecision. |
| Hu et al. (27) | Prospective design; clear inclusion/exclusion criteria; small but well-defined cohort. | Scans were done at baseline and 4 weeks post-treatment. Enough follow-up periods. | RECIST 1.1 used for short-term response; independent review; objective and reproducible. | Multivariable analysis performed; small sample limits adjustment; tumor site included. Not all confounders addressed. | Well-conducted prospective study but limited by small sample and potential overfitting. |
| Miao et al. (28) | Prospective; clear eligibility; consecutive enrollment; reasonable sample (n=28). | No lost to follow-up. Certain specific scan times. | Pathologic TRG (gold standard); blinded assessment; objective and clinically relevant. | Heterogeneous treatment regimens; no multivariable model for all confounders; small subgroups. | Strong prospective design but limited by sample size and regimen variability. |
| Arak et al. (29) | Retrospective; single-center; mixed cancer types; potential selection bias. | Reported enough follow-up times. | Overall survival (objective); no blinding but unlikely influenced. | Multiple cancer types; varied treatments; multivariable model used but residual confounding likely. | Retrospective design and heterogeneity; prognostic value suggested but not definitive. |
| Watanabe et al. (30) | Prospective, enrolled patients from two registered clinical trials. While it is a mixed cohort of various cancers, the source and recruitment method are clearly defined. | The study stated that the cutoff for follow-up, and the number of death events and censored patients is clearly reported. | OS is an objective, hard endpoint. The definition (from PET/CT scan to death/censoring) is clear and unlikely to be influenced by knowledge of the prognostic factor. | Performing univariate and multivariate Cox regression analyses, with adjustment. Sub-analyses: small sample sizes. Key clinical factors were not included in the multivariate models for the entire cohort. | Performing both univariate and multivariate analyses, adjusting for clinical and imaging variables. Tumor entity was included as a categorical variable. |
| Rong et al. (31) | Small sample size, single center. | The follow-up method and duration are not explicitly detailed for the entire cohort. The focus is on the 2-month response assessment. | Using RECIST criteria: standard, though not perfectly objective, endpoint. The correlation with IHC adds biological plausibility. | Presenting correlative and univariate analyses. No meaningful multivariate analysis was performed to adjust for other predictors. | Small sample size, no confounding adjustment, exploratory findings. |
| Zhang et al. (32) | Retrospective. Drawing from a single-center prospective database. Well-defined inclusion/exclusion criteria. | The follow-up protocol is clearly described, a defined cutoff date. The number of death events is high, and follow-up duration is reported. | PFS: while potentially subject to interpretation, was based on predefined, multi-modal criteria (imaging, biomarkers, clinical course). OS: an objective endpoint. | Extensive univariate and multivariate analyses, adjusting for wide array of potential confounders. | Primarily due to the retrospective design |
| Zheng et al. (33) | Consecutive patients with biopsy-confirmed cancer scheduled for treatment. | All patients completed NAC and surgery. | Not stating if pathologists were blinded to PET results. | Multivariate logistic regression used. | Confounding adjustment, complete follow-up. |
| Jiang et al. (34) | treatment-naïve LAOSCC patients. | all completed treatments. | nuclear medicine physicians blinded to clinical data. | small sample, no multivariate analysis. | small sample, no confounder adjustment |
| Qin et al. (35) | Newly diagnosed gastric cancer patients. | Median 12.5 months, mid-point imputation for missing death times. | Not specified. | Multivariate Cox regression, lasso regression, nomogram. | Confounding adjustment, enough follow-up. |
| Xu et al. (36) | Prospective, small sample size, single center. Considerable gender imbalance. | Patients were followed up every 2-3 months. However, the follow-up duration is not explicitly stated. The number of events (deaths) is not provided, making it difficult to assess the maturity of the survival data. | The outcome (OS) is an objective and robust endpoint. The PET analysis was performed by experienced physicians, though blinding is not explicitly mentioned | Survival analysis: performed based solely on PET positivity, without any adjustment for established prognostic factors in HCC such as tumor stage, liver function, AFP levels, etc. | True prognostic study with a survival endpoint. Failure to adjust for key confounding prognostic factors in the analysis. |
| Beyhan et al. (37) | Consecutive or clearly representative series of patients with gastric cancer. | Not clearly reported; follow-up duration and completeness not detailed. | OS and PFS clearly defined. | No multivariate analysis adjusting for clinical or pathological confounders. | No adjustment for confounders; unclear follow-up; small sample. |
| Zhang et al. (38) | Single-center, small sample, but well-defined inclusion criteria. | Follow-up clearly reported. | TRG (Evans grading), RFS, and OS clearly defined. | Multivariate analysis included FAPI parameters and clinical variables. | Small sample size; single-center design. |
| Ding et al. (39) | Prospective recruitment of patients with suspected localized PDAC. | Clear follow-up protocol; median follow-up of 17 months. | RFS and OS clearly defined and assessed. | Multivariate analysis performed including tumor size, differentiation, and FAPI parameters. | Prospective design, multivariate adjustment, clear outcome definitions. |
| Ding et al. (40) | Consecutive recruitment, single center. However, the final cohort was selected from 64 initially enrolled, with exclusions for unresectability. | Complete follow-up data. The follow-up is relatively short for OS analysis, and the median follow-up time is not provided for censored patients. | Objective and robust outcomes. Standard definition of RFS. Blinding of outcome assessors to PET parameters is not explicitly stated but is less critical for these hard endpoints. | Both univariate and multivariate analyses. Key confounders like stage, grade, and perineural invasion were included in multivariate. | Not representative of all PDAC patients. Short follow-up. |
| Zhao et al. (41) | A prospective study but analyzed retrospectively; selected from screened patients, including only those without metastases who underwent treatment. | The median follow-up was 24 months, which is adequate. All included patients were accounted for in the survival analysis. | PFS and OS are objective endpoints. RECIST 1.1 was used for progression, which is standard. Blinding of outcome assessors to FAPI-PET data is not mentioned. | Univariate and multivariate Cox regression were performed. Crucially, the multivariate analysis included clinical T and N stage. | Objective outcome assessment, confounding adjustment, enough follow-up. |
| Zhang et al. (42) | Small sample size. | All included patients were included in the analysis. The short-term follow-up for response assessment is adequate. | Treatment response was assessed using RECIST 1.1 by two imaging specialists blinded to clinical/PET results. | Logistic regression used. Small sample size and number of events limit the reliability of the multivariate, and susceptible to overfitting. | Highly specific patient group, not generalizable, not confounding adjustment. |
| Chen et al. (43) | Prospective, consecutive recruitment, single center, small sample size. | All patients completed NAC course, underwent scheduled PET scans, with pathologic assessment. No loss to follow-up. | Standard, objective pathological criterion. Pathologists’ blindness to the PET results, was not explicitly stated. Using of a definitive, clinically relevant endpoint minimizes bias. | Not any statement for confounding. | Prospective, representative of the patients. Objective assessment, enough follow-up. |
| Metzger et al. (44) | Retrospective, single center, small sample size. Clear inclusion criteria. | Not all patients underwent all follow-up scans; 52% had no follow-up FAPI PET, a subset had first follow-up scan. High rate of missing follow-up. | Based on the same FAPI-PET/CT and CT. Histopathological confirmation for up-/down-staged lesions was rarely obtained, and clinical follow-up/tumor markers are subjective surrogates. | Confounding adjustment using multivariate analysis. | Primarily due to significant attrition and the subjective, non-histopathological outcome assessment for many key findings. |
| Wu et al. (45) | Prospective; consecutive recruitment. Clearly defined inclusion/exclusion criteria; focusing on specific patient population. | Complete endpoints follow-up; substantial. Intention-to-treat basis analysis with clear documentation of events and censoring. | Objective, based on RECIST criteria assessed by follow-up imaging. PFS and OS are hard, unbiased endpoints. | Not any statement for confounding. | Prospective, enough follow-up, objective outcome assessment. |
| Jia et al. (46) | Single-center, small sample, selected from a phase II trial; may not represent broader PDAC population. | Median follow-up 9.4 months; 19/28 had progression; no loss to follow-up reported. | PFS/OS defined clearly; RECIST 1.1 used for response; blinded assessment not explicitly stated. | Multivariable analysis, but small sample limits adjustment. | Prospective design, clear PET/CT protocols, multivariable analysis. |
| Selcuk et al. (47) | Retrospective, single-center, heterogeneous GI cancers, mostly gastric. | Mean follow-up 15.0±11.2 months; no loss reported. | OS measured; PERCIST and RECIST used; blinded reading not explicitly stated. | No multivariable Cox model; unadjusted survival analyses; heterogeneous treatments. | Retrospective, no adjustment for confounders, heterogeneous cohort. |
